# Pathophysiological Effects of Contemporary Lifestyle on Evolutionary-Conserved Survival Mechanisms in Polycystic Ovary Syndrome

**DOI:** 10.3390/life13041056

**Published:** 2023-04-20

**Authors:** Jim Parker

**Affiliations:** School of Medicine, University of Wollongong, Wollongong, NSW 2522, Australia; jimparker@ozemail.com.au

**Keywords:** polycystic ovary syndrome, evolution, inflammation, insulin resistance, hyperinsulinemia, immune, infertility, endocrine disrupting chemicals, environment, microbiome, lifestyle, diet

## Abstract

Polycystic ovary syndrome (PCOS) is increasingly being characterized as an evolutionary mismatch disorder that presents with a complex mixture of metabolic and endocrine symptoms. The Evolutionary Model proposes that PCOS arises from a collection of inherited polymorphisms that have been consistently demonstrated in a variety of ethnic groups and races. In utero developmental programming of susceptible genomic variants are thought to predispose the offspring to develop PCOS. Postnatal exposure to lifestyle and environmental risk factors results in epigenetic activation of developmentally programmed genes and disturbance of the hallmarks of health. The resulting pathophysiological changes represent the consequences of poor-quality diet, sedentary behaviour, endocrine disrupting chemicals, stress, circadian disruption, and other lifestyle factors. Emerging evidence suggests that lifestyle-induced gastrointestinal dysbiosis plays a central role in the pathogenesis of PCOS. Lifestyle and environmental exposures initiate changes that result in disturbance of the gastrointestinal microbiome (dysbiosis), immune dysregulation (chronic inflammation), altered metabolism (insulin resistance), endocrine and reproductive imbalance (hyperandrogenism), and central nervous system dysfunction (neuroendocrine and autonomic nervous system). PCOS can be a progressive metabolic condition that leads to obesity, gestational diabetes, type two diabetes, metabolic-associated fatty liver disease, metabolic syndrome, cardiovascular disease, and cancer. This review explores the mechanisms that underpin the evolutionary mismatch between ancient survival pathways and contemporary lifestyle factors involved in the pathogenesis and pathophysiology of PCOS.

## 1. Introduction

There is general agreement that PCOS is a polygenic multisystem disorder arising from an interaction between genetic and environmental factors [1]. Comprehensive International Guidelines recommend a range of lifestyle-based interventions as first-line management for all women diagnosed with PCOS [2]. These recommendations are based on evidence that lifestyle therapies, such as diet and exercise, can control and reverse many of the biochemical and endocrine features of PCOS [2,3]. It has been hypothesized that contemporary lifestyle and environmental exposures are instrumental in the pathogenesis of PCOS due to a mismatch between our ancient and modern lifestyles and the environment [1,4,5,6].

PCOS affects 8–13% of reproductive aged women, is thought to be increasing in prevalence globally, and is estimated to affect up to 200 million women worldwide [6,7]. Women affected with PCOS present with a wide variety of symptoms (menstrual disturbance, acne, hirsutism, alopecia, subfertility, anxiety, and depression) that reflect the underlying multisystem pathophysiology [8,9,10]. Women with PCOS have an increased risk of pregnancy complications (deep venous thrombosis, pre-eclampsia, macrosomia, growth restriction, miscarriage, stillbirth, and preterm labour) [11], psychological problems (anxiety and depression) [12], and can progress to a range of other metabolic-related conditions (obesity, gestational diabetes, type two diabetes (T2DM), metabolic-associated fatty liver disease, chronic kidney disease, metabolic syndrome, cardiovascular disease, and cancer) [13,14,15,16]. The population-attributable risk of PCOS to T2DM alone has been estimated at 19–28% of women of reproductive age [17]. PCOS can be a progressive metabolic condition that makes a significant contribution to the chronic disease epidemic [18].

Genome-wide association studies (GWAS) have identified common PCOS risk alleles in women from Chinese and European populations, suggesting PCOS is an ancient inherited disorder that was present before humans migrated out of Africa [19,20]. Familial and twin studies [21,22], more recent Mendelian randomization [23,24], and transcriptome-wide association studies (TWAS) [25], also support a genetic basis for PCOS. From an evolutionary perspective, decades of research have characterized PCOS as an inherited polygenic trait that manifests after exposure to lifestyle and environmental risk factors [1,26,27]. Nevertheless, it is thought that genetic factors contribute less than 10% to disease susceptibility, as has been found with other lifestyle-related chronic diseases, such as obesity and T2DM [28]. The genetic basis of PCOS has previously been comprehensively reviewed and is not the focus of the current review [1,7,29]. 

The prime directive of all life is to optimize reproduction and species survival [30]. Reproduction and metabolism are intimately linked so optimal reproductive fitness requires optimal metabolism [31,32]. There is always an evolutionary trade off to optimize metabolism and/or reproduction, depending on the species and prevailing environmental conditions [33]. This is achieved by a complex network of hormonal and signalling molecules that link metabolism to reproductive cycles via hormonal regulatory processes, post-translational modification of enzymes, substrate-level inhibition of metabolic pathways, and epigenetic regulation of gene expression [34,35,36]. It has been hypothesized that PCOS may represent an evolutionary metabolic adaptation to balance energy substrate availability (glucose and fatty acid) and optimize reproduction [5]. This shift in focus to the importance of metabolic adaptation and dysregulation has also been emphasized in the International Guidelines [2,37]. A number of evolutionary hypotheses have been developed to try to explain the pathogenesis of PCOS and will be discussed in the following sections of this review [1,5,26,27,38].

A variety of lifestyle and environmental exposures have been found to contribute to the pathogenesis of PCOS [1,2,5,39]. These include diet and nutritional factors, exercise and sedentary behaviour, sleep and circadian disruption, endocrine-disrupting chemicals, stress, direct and indirect effects of climate change, and community support systems [1]. Contemporary lifestyle exposures are significantly different from the environmental conditions that existed throughout most of human evolution. Namely, starvation, predation, fear, increased maternal mortality, and exposure to different climatic conditions [4,27,40]. 

The reduction of chronic disease following lifestyle interventions, such as diet, exercise, and smoking cessation, has provided strong evidence for contemporary lifestyle as the primary “cause” of many diseases, including PCOS [41]. Optimal health and well-being are achieved when metabolic, cellular, and whole-body homeostasis is synchronized with the prevailing environmental conditions [34]. Multiple adaptive survival mechanisms (immune, metabolic, neurological, and hormonal) have evolved to ensure the re-establishment of homeostasis during periods of environmental and personal stress and work synergistically to maintain health [4,5,27,42,43]. Rapid social evolution has created a contemporary lifestyle and environment that is out of step with our evolutionary past [1,4]. Chronic disturbance to adaptive homeostatic regulatory networks, disrupt the “Hallmarks of Health” [44], resulting in maladaptive metabolic, immune, and physiological responses, that cause the observed features of PCOS [45,46].

**Figure 1:**
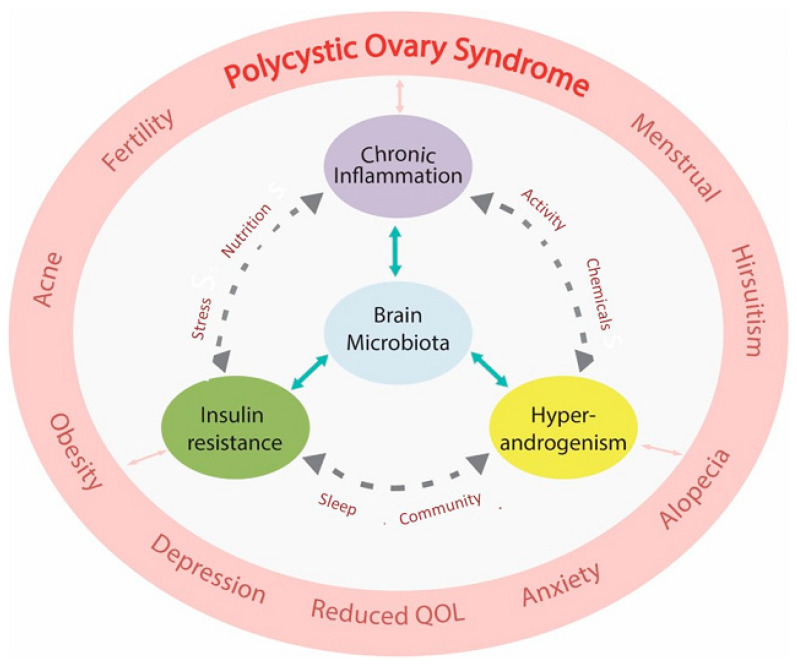
Pathophysiology of Polycystic Ovary Syndrome. Depicts the multi-directional interactions between nutritional and environmental lifestyle-related risk factors and the identified pathophysiological processes and symptoms in PCOS. Health is a result of the successful integration of multidimensional subcellular, cellular, and systemic, integrated circuits and networks. Disturbances in these networks due to dysbiosis, chronic inflammation, insulin resistance, and neuroendocrine deregulation in isolation or in combination can lead to loss of homeostatic resilience in the system. Combinations of adverse nutritional and environmental factors can disturb this network in a myriad of ways, at multiple different sites, and are responsible for the pathogenesis of PCOS. The influence of the exposome on developmentally programmed susceptibility genes programs the embryo/foetus to express the phenotypic manifestations of PCOS during childhood, adolescence, and adulthood, following exposure to lifestyle, dietary, and environmental factors. QOL, quality of life.

It is now appreciated that more than one pathophysiological mechanism is involved in the development of PCOS [47,48]. This narrative review outlines the relationship between lifestyle and environmental risk factors and the underlying pathophysiological mechanisms identified in women with PCOS. These include immune dysregulation (chronic systemic inflammation and oxidative stress), metabolic dysfunction (insulin resistance (IR), hyperglycaemia, and hyperinsulinemia), hormonal dysregulation (hyperandrogenism, estrogen, follicle-stimulating hormone, and luteinizing hormone), and gastrointestinal dysbiosis (decreased alpha diversity and increased gastrointestinal mucosal permeability) (Figure 1). The pathological processes are discussed in the context of the Evolutionary Model of PCOS [1].

## 2. Materials and Methods

The literature search focused on research publications related to the pathophysiology and pathogenesis of PCOS using the keywords listed above and related mesh terms for data on the evolutionary aspects of PCOS, chronic systemic inflammation, in-utero developmental epigenetic programming, insulin resistance, hyperinsulinemia, hyperandrogenism, reproductive changes, infertility, microbiome, dysbiosis, endocrine disrupting chemicals, lifestyle, diet, and physical activity. The databases searched included PubMed, Scopus, Cochrane, and Google Scholar. The literature has been searched repeatedly over the past 10 years. A glossary of abbreviations is included.

The present manuscript provides a summary of the pathogenesis and pathophysiology of PCOS in the context of the Evolutionary Model [1] and the Hallmarks of Health [44]. 

## 3. Chronic Systemic Inflammation

### 3.1. Evolution and the Advantages of a Proinflammatory Design

Inflammation is a normal physiological process that is an evolutionary conserved homeostatic mechanism in cells and tissues throughout the body [49]. Optimal health is achieved when a balance between pro- and anti-inflammatory processes removes aging, damaged or infected cells, and restores normal cellular function [50]. Inflammation is a protective mechanism in response to specific environmental conditions and occurs at a cost to normal tissue function [51,52]. Local anti-inflammatory mediators attempt to limit the systemic spread of inflammation and contain the inflammatory response. The resolution of inflammation is facilitated by the removal of the primary cause, local negative feedback loops, systemic regulation by the autonomic nervous system (ANS) [53], and glucocorticoids [44]. Chronic low-grade systemic inflammation can occur as a result of the failure of any of these homeostatic mechanisms and is a cornerstone of PCOS pathophysiology [54]. Large systematic reviews confirm the important role of chronic systemic inflammation in the pathogenesis of PCOS [8,46].

Rapid changes in the contemporary human environment have outpaced genetic adaptation, leading to a mismatch between our modern exposures, and selected metabolic and reproductive traits. This mismatch has resulted in a dysregulated inflammatory response that has increased susceptibility to many common chronic diseases, including obesity, type two diabetes, metabolic syndrome, cardiovascular disease, neuroinflammatory diseases, and PCOS [50]. Chronic low-grade inflammation or metaflammation, caused by poor-quality diet, nutritional excess, and other environmental factors, is maintained at a subacute level over long periods of time, enhancing inflammatory and metabolic signal transduction pathways that lead to the symptoms and diseases associated with PCOS [55]. The concept of metaflammation refers to the pathophysiological association between metabolic disorders and the immune system and is proposed to originate from the evolutionary crosstalk between immune and metabolic pathways [52]. 

The central role of chronic systemic inflammation and metabolic dysregulation and their relationship to evolution, obesity, the microbiota, endocrine-disrupting chemicals, and the recently described “Hallmarks of Health” are described in the following sections of this review. There is a particular focus on the molecular details and mechanisms involved in the pathophysiology of PCOS.

### 3.2. Overview of the Inflammatory Response

The human body has two parallel systems of cellular defence (innate and adaptive immunity) that work co-operatively to protect cells, individuals, and ultimately the species [56]. Billions of years of evolution have equipped unicellular organisms with a range of stress responses to abiotic environmental threats, such as temperature, salinity, sunlight exposure, heavy metals, and oxygen [57]. In addition, multicellular organisms possess elaborate innate and adaptive immune responses to defend against exposure to non-infectious (reactive oxygen species, uric acid, cholesterol, microparticles, and exosomes) and infectious agents (bacteria, viruses, protozoa, parasites, fungi) [58]. These responses are activated by a range of biological mechanisms, including oxidative stress and reactive oxygen species (ROS), advanced glycation end-products (AGE), and via pattern recognition receptors (PRR) [59].

#### 3.2.1. Oxidative Stress in PCOS

The evolution of life chemistry and metabolism dates back 3.5 billion years [60]. The first cellular life forms arose in an anaerobic environment and most of the pathways of intermediate metabolism (glycolysis, fatty acid synthesis and oxidation, pentose phosphate pathway, Krebs cycle, electron transport, and many more), developed in an environment where oxygen was toxic [61]. Approximately 2.4 billion years ago, cyanobacteria started producing oxygen from photosynthesis, raising the atmospheric oxygen to 2–4%. A billion years later, during the Pre-Cambrian period, oxygen levels rose, and multicellular organisms flourished. This resulted in the ability of cells to use oxygen to make adenosine triphosphate (ATP) and produce ROS (superoxide, hydroxyl radical) as a metabolic by-product, and for the purposes of cell signalling and defence, and detoxifying oxygen with a variety of antioxidant systems [62]. ROS contain an unpaired electron that makes them extremely unstable and reactive. ROS attempt to stabilize themselves by scavenging electrons from healthy cells and cause oxidative damage. 

Living cells can be differentiated from dead cells because of the cessation of the coordinated flow of energy that occurs due to electron transfer from one molecule to another during metabolism, following failure of adaptive responses to restore cellular homeostasis [63]. When the flow of electrons to the mitochondrial electron transport chain is disrupted by environmental factors, such as microbial infection, chemical toxins, accumulation of metabolic intermediates, due to nutritional excess, and other cellular stressors, metabolic mismatch occurs [62]. Electrons are diverted away from mitochondria, mitochondrial oxygen consumption falls, and cytoplasmic oxygen rises. This redox imbalance creates ROS and reactive nitrogen species (RNS) that initiate innate immune responses designed to defend and protect the cell [64]. Mitochondrial structure, dynamics, biogenesis, and membrane potential are altered in women with PCOS [65].

The body has an in-built system of antioxidants to stabilize and neutralize ROS and protect the cell. Antioxidants are highly stable molecules that have the unique ability to serve as electron donors to help stabilize free radicals without becoming reactive themselves. Oxidative stress refers to the imbalance between the production of oxidant species and antioxidant defences, and the generation of excessive amounts of ROS that underlie the various forms of cell death [66]. Oxidative stress can arise from endogenous (leakage of ROS from mitochondrial oxidative phosphorylation, cytochrome P-450 detoxification enzyme systems, peroxisomal oxidases, and nicotinamide dinucleotide adenine phosphate oxidases) or exogenous sources (environmental chemicals, cigarette smoke, alcohol, ionizing radiation, microbial infection, stress, and sleep deprivation) [66,67] and is a potent stimulator of inflammation [68,69]. Oxidative stress has been found to play a central role in the pathogenesis of PCOS [66]. Oxidative stress can impair insulin signalling and cause IR [70] and dysregulate follicular calcium which results in reproductive and menstrual dysfunction [71], oxidize plasma proteins that may act as pro-inflammatory mediators [72], cause lipid peroxidation [73], and induce DNA damage [74] in women with PCOS.

Cumulative studies show an association between oxidative stress and PCOS [64,66]. In addition, oxidant and antioxidant status has been found to vary between individuals because of differences in diet, lifestyle, and enzymatic and dietary antioxidants [64,68]. A recent case-control study showed that plant-based dietary pattern is associated with a lower odds ratio of PCOS and suggested that antioxidant-rich foods may protect the body against oxidative damage [75]. Dietary total antioxidant capacity was subsequently assessed using the Nutrient Data Laboratory of the United States Department of Agriculture reference values. The investigators reported a significant reduction in the odds of PCOS in women that consumed a high total antioxidant containing plant-based diet [76]. These findings support the existing body of dietary pattern research that recommend healthy diets for the management of PCOS [2,77].

#### 3.2.2. Advanced Glycation End Products and PCOS

Advanced glycation end-products are reactive molecules that are formed by non-enzymatic reactions of carbohydrates with proteins, lipids, or nucleic acids [78]. Advanced glycation end products result in the irreversible cross-linking of proteins and loss of protein structure and function and can initiate apoptosis [79]. Advanced glycation end products can be generated endogenously under normal conditions, and can be ingested in food, particularly a cooked fast-food diet, and with cigarette smoking [80]. Advanced glycation end products can cause oxidative stress and inflammation, resulting in cellular and tissue damage when produced or ingested in excessive amounts [81]. Protective circulating anti-inflammatory receptors called soluble receptors for advanced glycation end products and membrane-bound receptors of advanced glycation end-products (RAGE) are associated with protection against AGE [82].

The interaction of AGE with their membrane receptors activates intracellular signalling pathways that lead to increased oxidative stress, inflammation, IR, diabetes, hyperandrogenism, obesity, and ovulatory dysfunction, all of which have been associated with PCOS [83,84]. Recent data have shown elevated circulating levels of AGE and increased expression of RAGE receptors in ovarian tissue [85,86]. Proinflammatory AGE–RAGE signalling has been found to cause altered steroidogenesis and follicle development in ovarian granulosa cells in PCOS [87,88]. In addition, hyperandrogenism induces endoplasmic reticulum stress in granulosa cells, resulting in increased accumulation of AGE in the ovary [86].

Modern Western diets are rich in AGE which is absorbed through the intestine [84]. A high-glycaemic diet and excessive glucose ingestion result in elevated blood glucose levels and the generation of AGE that bind with cell membrane-bound RAGE and activate inflammation [89,90]. Ligand binding by glycated proteins and lipids to RAGE stimulates intracellular signalling events that activate nuclear factor kappa-light-chain-enhancer of activated B (NF-κB). Nuclear factor kappa B controls several genes involved in inflammation, and RAGE itself is upregulated by NF-κB, establishing a positive feedback cycle that leads to chronic inflammation [90].

Studies have demonstrated that the intake of the low-AGE containing diet is associated with favourable metabolic and hormonal profiles as well as fewer oxidative stress biomarkers in patients with PCOS [91]. One study employed a low-AGE diet, consisting of Mediterranean-style foods cooked at temperatures below 180 degrees by boiling, poaching, stewing, or steaming [91]. High-temperature cooking above 220 degrees Celsius by roasting, grilling, and baking was avoided. Dietary recommendations for minimising ingestion of AGE includes increasing consumption of a whole foods that include vegetables, fruits, seafood, and whole grains while reducing the consumption of high AGE containing foods. These include highly processed foods (packaged meats, cheese, and snack foods), excessive sugar in sweets and beverages, and fried foods [77,84]. Adoption of other healthy lifestyle behaviours, such as exercise, maintaining normal body weight, and cessation of tobacco consumption, are also important for reducing AGE [84,92].

#### 3.2.3. Pattern Recognition Receptors and the Innate Immune System

The existence of receptors expressed by innate immune cells that were responsible for detecting microbial products was first proposed by Charles Janeway in 1989 [93]. Polly Matzinger subsequently proposed the “Danger Theory”, suggesting that the immune system produced molecules that initiate and propagate inflammation in response to tissue stress, damage, or infection [94]. In 2013, Robert Naviaux further expanded this concept to the “Cell Danger Response” (CDR) [95]. Naviaux proposed that evolutionary selection has preserved a similar response to a variety of threats as cells have a limited number of ways they can mobilize existing cellular machinery and energy. The CDR is, therefore, an evolutionary-conserved cellular protective response that is activated when a cell encounters a chemical, physical, or microbial threat that could injure or kill the cell [95]. More recent research has characterized the molecular details of an elaborate danger detection system, involving PRR, damage-associated molecular patterns (DAMPS), pathogen-associated molecular patterns (PAMPS), inflammasomes, and an intricate system of signalling molecules that activate a system-wide network of innate and adaptive immune responses [59]. This has been called the “Integrated Stress Response” and is reviewed in detail in previous reports [96,97].

There are five types of PRR that can be classified into two main groups based on their cellular localization [98]. Toll-like receptors (TLR) and C-type lectin receptors (CLR) are transmembrane receptors that allow sensing of host-derived DAMPS and PAMPS at the cell surface or within membrane-bound intracellular compartments. Specific cytoplasmic-based receptors also provide an intracellular recognition system for sensing DAMPS and PAMPS. Pattern recognition receptors are also excreted extracellularly and can be found in interstitial fluid and the bloodstream, where they play an important role in pathogen recognition [59]. Pattern recognition receptors also activate multiple types of cell death pathways, such as apoptosis and pyroptosis (a rapid pro-inflammatory form of cell death), if cellular defence against PAMPS and DAMPS is unsuccessful [52,59]. 

There are three main types of molecules involved in signal transduction following infectious or danger-related ligand binding to PRR: protein kinases, adaptor proteins, and transcription factors [98]. Signal transduction occurs via several common pathways, including NF-κB, mitogen-activated protein kinase (MAPK), and inflammasomes [52,98]. The signals they generate can crosstalk with each other and can converge into several common pathways. Each of the PRR initiate signalling cascades that cause epigenetic modulation of gene expression and posttranslational modification of cytokine precursors. This results in activation of the innate immune response and leads to degradation of microbes, production of inflammatory cytokines, and recruitment of the adaptive immune response [59,99]. Once PAMPS and DAMPS bind with TLR and nod-like receptors, they activate the formation of inflammasome complexes that form an essential element of the innate immune response [52,100].

Unlike adaptive immunity, innate immunity does not recognize every possible antigen. Innate immunity recognizes PAMPS shared by related groups of microbes that are not found in mammalian cells, such as lipopolysaccharide (LPS) from gram-negative bacterial cell walls. This early induced innate immunity (4–96 h) involves the formation of inflammasomes that lead to the release of chemokines and recruitment of defence cells. The recruited defence cells include phagocytic leukocytes, such as neutrophils, eosinophils, and monocytes and tissue phagocytic cells, such as macrophages, macrophages, and mast cells, that release inflammatory mediators and basophils, eosinophils, and natural killer cells [59].

Pattern recognition receptors represent evolutionarily conserved pathogen and damage recognition mechanisms that constitute the starting point for the inflammatory response. These cell-autonomous stress responses have evolved to form the basis of the non-antigen-dependent defence mechanisms that characterize innate immunity [94,101]. Lipopolysaccharide released by gram-negative bacteria in the dysbiotic gastrointestinal microbiome binds with TLR on sub-mucosal macrophages, resulting in the activation of NF-κB and inflammatory cytokine production and secretion [102]. This mechanism is thought to be a major contributor to chronic inflammation in women with PCOS and plays a significant role in the pathogenesis (discussed in Section 6) [54,103].

#### 3.2.4. Inflammasomes in PCOS

Inflammasomes are multiprotein self-assembling complexes in the cytoplasm that form an integral part of the innate immune response [104]. They are produced in response to a variety of danger signals and are also involved in cellular apoptosis and pyroptosis [52]. Inflammasomes act as finely tuned alarm systems that trigger and amplify innate defence mechanisms in response to cellular stresses and infection [100]. The inflammasome complex contains a sensor molecule, an adaptor protein, and a pro-inflammatory caspase-1 enzyme [104]. Once activated by DAMPS (such as LPS) and PAMPS, the inflammasome complex converts procaspase-1 to the active caspase-1 enzyme which subsequently activates pro-inflammatory cytokines (IL-1B, IL-18) that are released into tissues and circulation [105].

Inflammasomes form an integral part of the common CDR pathway for cellular protection from multiple types of threatening stimuli [51,94]. Interruption to the flow of electrons in metabolism, generation of ROS, and other mechanisms discussed above, activate innate intracellular defence mechanisms in an attempt to contain and eliminate cellular threats [62]. The CDR also includes the release of purinergic signalling to neighbouring cells and immune cells that activate inflammation [106]. If the threat is contained, the CDR resolves and normal cellular function is restored [51]. If the CDR is unsuccessful, pyroptosis pathways are activated and the cell is sacrificed in a further attempt to contain the threat and protect the organism [52]. If the inciting stimulus persists, activation of chronic inflammation can result in tissue damage and disease, such as PCOS.

Inflammasomes and their pro-inflammatory cytokines and chemokines have been investigated for their role in inflammation, oxidative stress, ovulation, fertilization, steroidogenesis, glucose metabolism, IR, and adipogenesis and may be involved in the pathogenesis of PCOS [100,107,108]. These and other inflammatory mediators, such as adipokines (leptin, adiponectin, vaspin, resistin, visfatin, and omentin-1), cyclophilin A, vascular dysfunction mediators (endothelin-1, vascular cell adhesive molecule-1), NF-κB, and epigenetic regulators (microRNAs) are thought to have a role in the pathogenesis of PCOS and have recently been reviewed [105].

#### 3.2.5. Adaptive Immune Response in PCOS

The adaptive immune system involves antigen-specific defence mechanisms that are designed to react to and remove specific antigens [109]. The adaptive system involves humeral and cell-mediated immunity and may take several days to become effective. The body recognizes an antigen as foreign when epitopes (fragments of an antigen that react with antibodies or lymphocyte receptors) bind to specific receptors on the surface of B-lymphocytes and/or T-lymphocytes [110]. It is estimated that the human body can recognize 10^7^ epitopes and make up to 10^9^ different antibodies [111]. Nevertheless, activation of both the innate and adaptive immune systems was primarily designed to be acute and short-lived in order to contain and eliminate a multitude of environmental threats.

The majority of the research on the role of adaptive immunity in PCOS has been conducted on T-cells and their subpopulations [109]. T-cells play a crucial role in mediating inflammation and IR by secreting proinflammatory cytokines [112,113]. T-cells promote follicle development and selection by releasing specific chemokines and growth factors and producing cytotoxic signals to induce apoptosis of granulosa cells [114]. Available evidence suggests that there may be a general decline in adaptive immunity and regulatory T-cell function in PCOS [109]. Recent studies have suggested that immune system dysregulation, including T-cell dysfunction [115,116], may play a role in the pathogenesis of PCOS [117].

The goal of the innate and adaptive immune response is to provide a balance between pro- and anti-inflammatory tissue turnover and protective measures, in order to achieve whole-body inflammatory homeostasis [118]. As is the case with metabolism, the internal inflammatory response needs to be matched to prevailing external environmental conditions [34]. The evolutionary theory of PCOS proposes that there is a mismatch between our evolutionary conserved metabolic and immune signalling systems and our modern environment [1,119,120]. Accumulating evidence over the past 25 years suggests that systemic inflammation is modulated by neural and humoral communication pathways that connect the brain to the immune system [121,122]. Neuroimmunomodulation is a way for organisms to regulate whole-body inflammatory homeostasis and optimize survival [118]. This interconnected regulatory system has evolved over millions of years of evolutionary pressure from exposure to pathogens and other environmental threats.

### 3.3. Neuroimmunomodulation and the Link between the Nervous System and PCOS

Both the immune and nervous systems share many similarities and have unique qualities that allow them to sense changes in the internal and external environments and counteract deviations in homeostasis [118]. Communication between the nervous, endocrine, and immune systems involves evolutionary-conserved mechanisms that are essential for host defence and survival [121]. The immune system and ANS can respond to numerous common regulatory molecules, including cytokines, neurotransmitters, and glucocorticoids [123]. The brain is the ultimate regulator of whole-body homeostasis of all physiological parameters. This includes blood glucose, thermoregulation, hydration, electrolyte levels, blood pressure, stress responses, feeding, behaviour, reproduction, body weight, and whole-body inflammatory balance [34,48].

Regulation of the inflammatory response has previously been thought to be autonomous [124]. Substantial evidence now suggests that the nervous system exerts an active role in maintaining inflammatory homeostasis [48,125,126]. The brain participates in a bidirectional network of mediators, including hormones, cytokines, and neurotransmitters, that monitor, coordinate, and regulate the systemic inflammatory response [121,126]. The magnitude of the inflammatory response is crucial to adaptation and survival. An inefficient response can result in immunodeficiency, infection, and cancer, and an excessive response can lead to morbidity and mortality [127]. Abnormalities in the neuroendocrine-immune response are implicated in the pathogenesis of many chronic diseases, including obesity, atherosclerosis, autoimmune disease, depression, and PCOS [48,109,122,123].

#### 3.3.1. Anatomy of Neuroendocrine-Immune Connections

**Figure 2:**
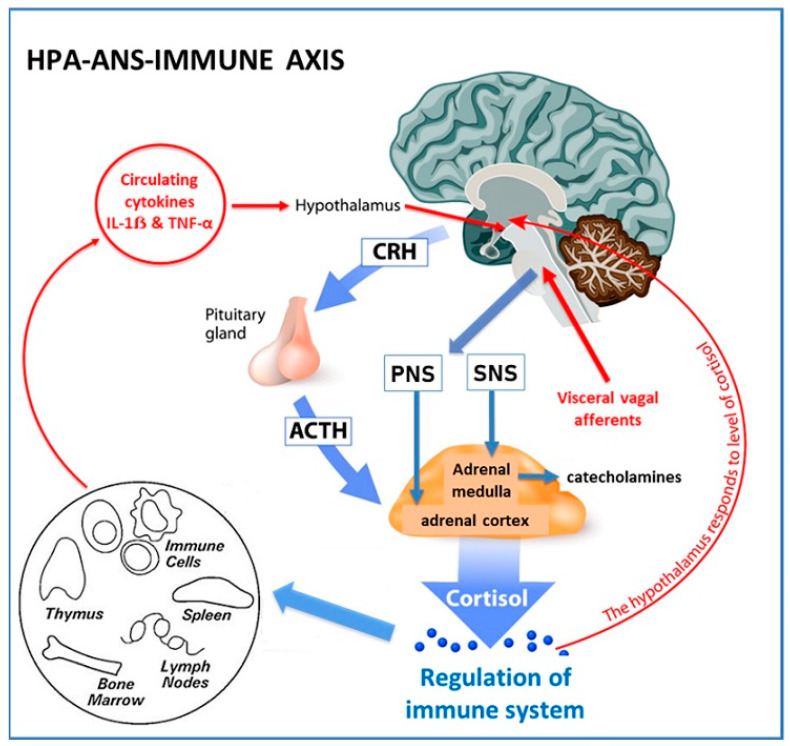
Hypothalamic-Pituitary-Adrenal-Immune Axis (HPA). The hypothalamus releases corticotropin releasing hormone that stimulates production of adrenocorticotropic hormone (ACTH) from the anterior pituitary. ACTH stimulates the synthesis of immunosuppressive glucocorticoids (cortisol) from the adrenal cortex [133]. Pro-inflammatory cytokines and neural inputs activate the HPA-axis to release ACTH, and the HPA-axis is subject to a classic negative feedback loop by cortisol that inhibits both corticotropin releasing hormone and ACTH [136]. Sympathetic neural activation of chromaffin cells in the adrenal medulla leads to an increased release of catecholamines into the circulation. Sympathetic innervation of cortical cells leads to the release of glucocorticoids. CNS-controlled SNS output is, therefore, converted to hormonal immunoregulation in peripheral tissues. ANS, Autonomic Nervous System; Parasympathetic Nervous System (PNS); Sympathetic Nervous System (SNS); CRH, Corticotropin Releasing Hormone; Adrenocorticotropic Hormone (ACTH); IL-1ẞ, interleukin-1ẞ; TNF-α, tumour necrosis factor-α. © Designua|Dreamstime.com.

Animal and human research over the past six decades have investigated the extensive network of afferent and efferent communication mechanisms that coordinate the systemic inflammatory response [121,122,128]. There is now a consensus that “the inflammatory reflex” is composed of (1) a system of sensors (that identify PAMPS and DAMPS), (2) an afferent arm which conveys information about systemic inflammatory status to the central nervous system (CNS), (3) processing centres in the brain that integrate and interpret incoming signals (hypothalamic nuclei and brain stem autonomic neurons), (4) and an efferent arm which exerts immunomodulatory functions (Hypothalamic-Pituitary-Adrenal (HPA)-axis, ANS) [125]. The brain exerts strong immunomodulatory effects on a variety of components of the immune system by activation of the HPA-axis and ANS (Figure 2).

The hypothalamus plays a central role in sensing and coordinating neural and humoral factors and can modulate inflammatory pathways [48]. Circulating cytokines, such as IL-1ẞ and Tumour Necrosis Factor-alpha (TNF-α), can cross the blood–brain barrier by a carrier-mediated mechanism [129] or via the circumventricular organs [130]. The hypothalamus can also receive input from visceral vagus afferent fibres after they synapse with the dorsal motor nucleus of the vagus nerve in the brainstem. This has been termed the “cholinergic anti-inflammatory pathway” [121]. Ascending connections reach the hypothalamus via the nucleus tractus solitarius [131]. The effector arm or efferent system is modulated by the HPA-axis and the sympathetic (SNS) and parasympathetic (PNS) components of the ANS [121,132].

The HPA-axis is a neurohormonal pathway that has classically been studied for its role in regulating the immune system [133] (Figure 2). The SNS modulates both pro- and anti-inflammatory activities [121]. The SNS innovates primary (thymus and bone marrow) and secondary (spleen, lymph nodes, and tissues) lymphoid organs. Sympathetic neurons release noradrenalin and adrenalin that interact with adrenoreceptors on lymphocytes and macrophages and stimulate the production of cytokines that result in anti-inflammatory effects [134]. The PNS also plays a significant role in modulating immune cells and inflammatory activity [121]. Evidence that vagal afferent fibres relay messages to the CNS that inflammation is present in other body sites has been demonstrated in animal models, although evidence for the mechanisms of activation is still not clear [122]. The parasympathetic nervous system has an anti-inflammatory effect through release of acetylcholine that interacts directly with nicotinic receptors on macrophages [135], and also indirectly with the spleen [122]. Overall, the SNS and PNS appear to act synergistically to downregulate inflammation.

#### 3.3.2. Neuroimmunomodulation in PCOS

Several studies have examined the bi-direction connections between the nervous and immune systems in PCOS. Women with PCOS have been found to have increased SNS activity by muscle and skin microneurography, heart rate variability, measurement of nerve growth factor, and catecholamine metabolites [126,137]. Insulin has been found to stimulate SNS output, and there is a complex bidirectional relationship between IR and sympathetic activity in both the hypothalamus and ovary [137]. Although most of the research has focused on SNS overactivity, reduced PNS activity has also been demonstrated [132]. Impaired ANS function has been proposed as a contributor to hyperandrogenemia via mechanisms in the hypothalamus, adipose tissue, and ovary [138,139].

SNS activity can be reduced in women with PCOS by electroacupuncture, treatment of obstructive sleep apnoea with positive airway pressure, renal denervation in refractory hypertension, pharmacotherapy, exercise training, and weight loss [137,140]. Taken together, these data suggest that an imbalance of the ANS is likely to play a reversible role in the pathophysiology of PCOS.

### 3.4. Hyperandrogenism and Chronic Inflammation

Chronic systemic inflammation can cause hyperandrogenism [46] and elevated androgens can affect immune cells, resulting in predominately anti-inflammatory effects on the immune system [141]. There is debate in the literature regarding the direction of causation, physiological importance, and evolutionary significance of these processes. The preponderance of the evidence and the most widely accepted current view is that hyperandrogenism is secondary to the synergistic actions of chronic systemic inflammation and IR through upregulation of ovarian theca cell androgen synthesis (discussed in Section 4.5) [142]. The evidence presented in the current review also supports this paradigm.

Serum androgens can be derived from multiple tissues including the adrenal gland, adipose tissue, and ovaries [143]. Most serum androgens in PCOS are thought to be produced by the ovaries. Nevertheless, it is likely that multiple mechanisms are involved in excessive androgen production in different tissues in women with PCOS [143,144]. Low-grade chronic systemic inflammation is commonly found in patients with PCOS exhibiting hyperandrogenism, and ovarian inflammation and fibrosis are part of the histopathology [145]. Ovarian inflammatory mechanisms have been linked to systemic markers of inflammation (CRP and white cell count) [144,145,146] independent of body mass index (BMI) [147]. An extensive range of inflammatory cytokines have been associated with impaired folliculogenesis and androgen synthesis in women with PCOS [144]. Cytokines can influence the inflammatory response by epigenic mechanisms that alter gene expression or by posttranscriptional regulatory processes [46]. Oxidative stress has been reported to enhance ovarian steroidogenic enzymes and increase androgen levels in women with PCOS [67]. Endoplasmic reticular stress pathways are activated in the ovaries in mouse models and in humans and may contribute to the pathophysiology of PCOS through multiple effects in granulosa cells [148,149]. 

As discussed previously, AGE and RAGE have been found to cause altered steroidogenesis in granulosa cells in PCOS [87]. Dehydroepiandrosterone is a circulating pre-androgen produced in the adrenal cortex that has been found to stimulate inflammation and impair ovarian function in PCOS [150]. Increased luteinizing hormone (LH) levels, commonly found in PCOS, can amplify the abnormalities described in theca cell steroidogenesis [105]. The combined effects of these processes result in excess follicular androgens which combine with increased levels of insulin to downregulate aromatase levels in granulosa cells. This creates a continuous feedback loop between inflammation, IR, and hyperandrogenism with no apparent beginning or end. Despite these uncertainties, diet-induced inflammation can invoke hyperandrogenism [142], and effective treatment of inflammation can normalize androgen levels and restore fertility in women with PCOS [151]. In summary, accumulating evidence suggests that the ovaries are not the primary cause of hyperandrogenism in most women with PCOS (see Section 4.5).

The influence of androgens in the pathogenesis and pathophysiology of PCOS is undisputable, despite the debate regarding the primary mechanism of causation. Data derived mostly from animal models of PCOS clearly indicate that intrauterine exposure to elevated androgen levels induces the development of PCOS traits in adult females. PCOS can also develop in women with other hyperandrogenic syndromes, such as congenital adrenal hyperplasia, lipodystrophy, and ovarian or adrenal tumours. In addition, there has also been debate regarding the evolutionary “advantage” of hyperandrogenism in PCOS. Inflammation-induced hyperandrogenism has been proposed as a possible compensatory mechanism to restore homeostasis within the immune system given the anti-inflammatory effects of androgens in women [46]. It is also possible that hyperandrogenism (due to inflammation and/or hyperinsulinemia) may represent an adaptive physiological mechanism to down-regulate reproduction during periods of environmental (infection, starvation, and climatic) and personal stress. In addition, there may be other individual advantages, such as increased strength and fitness [1].

### 3.5. Summary of the Role of Inflammation in PCOS from an Evolutionary Perspective

A significant body of the literature supports the role of chronic inflammation in the pathogenesis of PCOS. Billions of years of evolution have constructed a cooperative system of sensors, receptors, hormones, cytokines, chemokines, and other signalling molecules that invoke intracrine, autocrine, paracrine, and neuroendocrine mechanisms designed to regulate cellular, tissue, and whole-body inflammatory responses. This process forms part of the repertoire of adaptive survival responses that are intimately connected with total body metabolic homeostasis to ensure *individual* survival. Inflammation invokes a series of adaptive metabolic survival mechanisms, such as IR, to ensure adequate energy supply to immune cells. Neuroendocrine mechanisms act as a counter-regulatory anti-inflammatory mechanism to control inflammation and restore homeostasis. In addition, immune and metabolic physiology are intimately linked to reproduction, to optimize fertility and ensure *species* survival. As a result, both chronic inflammation and IR can contribute to hyperandrogenemia, which also has adaptive survival advantages that restore immune homeostasis and temporarily downregulate reproduction.

## 4. Insulin Resistance and Reduced Insulin Sensitivity

### 4.1. Physiological Actions of Insulin

Insulin is a peptide hormone (51 amino acids) produced by the beta cells in the islets of Langerhans in the pancreas where it is stored in cytoplasmic granules as an inactive hexamer with zinc and calcium [152]. Insulin release is characterized by low-level basal secretion in the fasting state and an oscillating pulsatile ultradian pattern (1–2-h cycle), when stimulated. Insulin binds with its receptor which is a large (320 kDa) transmembrane protein with an intracellular domain that acts as a tyrosine kinase. The insulin receptor is highly conserved from an evolutionary perspective, and is present in all mammalian cells, although the distribution is unequal (40 per red blood cell, 300,000 per adipocyte) [153]. Insulin binds to the alpha-subunit and induces autophosphorylation of specific tyrosine residues on the cytoplasmic side of the membrane [43,154]. The activated insulin receptor initiates signal transduction via the phosphatidylinositol-3 kinase (PI-3K) metabolic pathway and the mitogen-activated protein kinase pathway (MAPK) which is involved in cell growth and proliferation [154]. There are multiple physiological stimulators (vagal PNS, growth hormone, chronic cortisol, prolactin, and gonadotropins) and inhibitors of insulin (adrenaline, noradrenaline, SNS, parathyroid hormone, somatostatin, acute cortisol, and pancreatic polypeptide) [155]. Glucose is by far the most potent stimulator of insulin and all the others are regulatory and much less effective.

Insulin has pleiotropic metabolic and growth-promoting anabolic effects throughout the body [155]. Importantly, the cellular effects of insulin are tissue specific, and not all cells require insulin to transport glucose into cells. Insulin signalling increases the availability of GLUT-4 glucose transport proteins to the surface of insulin-dependent cells (skeletal and cardiac muscle, adipose tissue, and vascular endothelium). The liver and brain are insulin-independent and use other GLUT transporters in addition to using insulin-responsive GLUT-4 transporters. Insulin modulates a wide range of tissue-specific physiological processes in addition to facilitating glucose removal from the blood [34]. Insulin enhances fat storage and inhibits lipolysis in adipose tissue, stimulates glycogen synthesis in muscle and liver, inhibits hepatic glucose output (gluconeogenesis), causes vasodilation in vascular endothelium, and the heart (via production of nitric oxide) and enhances sodium reabsorption in the kidney [155,156]. 

Insulin also has a direct anti-inflammatory role by preventing hyperglycaemia-related generation of ROS and AGE, inhibiting NF-kB (by reducing the production of inflammatory cytokines), inducing vasodilation (via nitric oxide release), reducing leukocyte adhesion to the endothelium [157], and by inhibiting formation of the NLR inflammasome complex [158]. As a result, hyperglycaemia and IR are pro-inflammatory states. Insulin resistance can cause inflammation, and inflammation can cause IR in women with PCOS [157,159]. Most of the literature has focused on the evolutionary benefit of the pro-inflammatory effects of IR in starvation, trauma, and infection (see Section 4.4) [159]. In contrast, the anti-inflammatory effects of insulin may have an evolutionary protective effect against excessive activation of the systemic immune response following exposure to small amounts of ingested antigens in a similar manner to the gastrointestinal mucosal immune system [160,161]. 

In summary, insulin’s overall role is to control energy conservation and utilization during feeding and fasting states [152]. Insulin acts as a metabolic switch between anabolic and catabolic processes to regulate blood glucose levels and has a significant anti-inflammatory effect. Insulin provides a direct link between metabolism and immune regulation and is paramount in activating numerous adaptive survival mechanisms [162]. Dysregulation of the protective physiological functions of insulin is instrumental in the pathogenesis and progression of many chronic diseases, including PCOS [152,163,164]. 

### 4.2. Reduced Insulin Sensitivity versus Insulin Resistance in PCOS

Diminished tissue sensitivity to insulin has become characterized as a pathological condition known as IR as a result of the association between IR, metabolic conditions, and chronic disease [17,164]. Being able to vary the sensitivity of the cellular and tissue response to insulin is an evolutionarily conserved protective mechanism used by many species (insects, worms, vertebrates, and humans) to enhance survival [165]. Insulin resistance is a categorical variable that has an arbitrary definition in research studies but has no agreed definition or normal range in clinical practice [2,166]. Reduced insulin sensitivity is a continuous variable that is considered a quantitative trait (interaction of multiple genes with the environment that results in a continuous distribution of phenotypes), in evolutionary medicine [167].

The gold standard for assessing IR is the hyperinsulinemic-euglycemic clamp (clamp) test. The clamp test is performed using insulin and glucose infusions and is time-consuming, labour-intensive, expensive, and has associated risks [168]. A high-dose insulin infusion (80 mU/m^2^/min) is used to suppress hepatic gluconeogenesis and create a steady-state blood glucose concentration. The rate of glucose infused, in order to maintain a steady state, is equal to whole-body glucose disposal. Decreased insulin sensitivity is defined as IR when the glucose disposal rate is less than an arbitrary cut off of 4.45 mg/kg/min [168].

It is important to distinguish between reduced insulin sensitivity and IR as most women with PCOS have reduced insulin sensitivity, but not all women with PCOS meet the experimental criteria for insulin resistance. The reported prevalence of IR in PCOS has varied widely due to the heterogeneity of diagnostic criteria for PCOS, the variety of assessment methods, and the arbitrary definition of IR selected for different studies [169]. Nevertheless, IR has been considered a central feature in the majority of women with PCOS [170]. A systematic review of hyperinsulinemic-euglycemic clamp studies found that women with PCOS have a 27% reduction in insulin sensitivity compared to matched controls [163].

When considered from an evolutionary perspective, women with a PCOS phenotype would have improved survival chances during times of increased physiological demand or imposed environmental stress but be more vulnerable to the pathological effects of IR when exposed to contemporary lifestyle factors [1,5]. When viewed as a continuous variable, it is likely that all women with PCOS, whether obese or lean, have reduced insulin sensitivity [163,171,172].

### 4.3. Mechanisms of Insulin Resistance in PCOS

Insulin resistance can be caused by numerous mechanisms, including hyperinsulinemia, insulin receptor variants, receptor antagonists and agonists, autoantibodies, oxidative stress, advanced glycation end-products, hormones, nutrient sensors, inflammatory cytokines, and metabolic intermediates [155,173]. It is likely that more than one mechanism is involved in any individual, given the interactive nature of many of the pathophysiological processes and signalling pathways.

**Figure 3:**
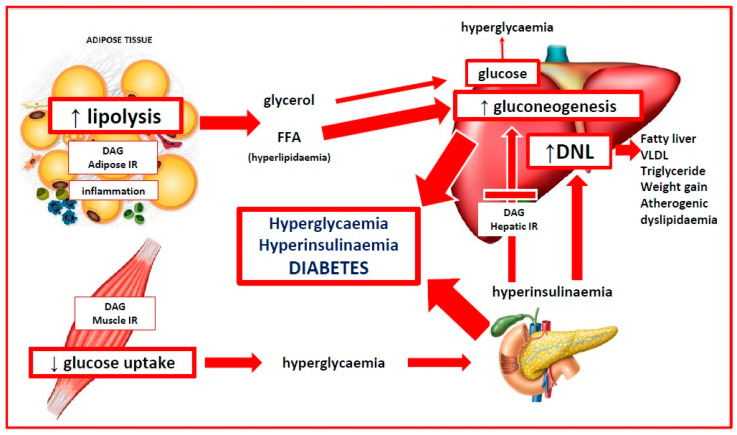
Pathophysiology of insulin resistance. Any of the causes listed above can impact insulin signalling pathways and lead to tissue-selective impairment of insulin action. Once insulin resistance occurs in muscle, glycogen synthesis and glucose uptake from the circulation are reduced. Since muscle constitutes approximately 50% of body mass, insulin resistance in muscle makes a significant contribution to hyperglycaemia. Insulin resistance in adipose tissue leads to impaired lipogenesis and continued lipolysis, with release of glycerol and free fatty acids into the circulation. Hepatic insulin resistance impairs glycogen synthesis and prevents insulin from inhibiting gluconeogenesis. Adipose lipolysis supplies substrates for continued hepatic gluconeogenesis. Together, the effects of decreased muscle glucose uptake, adipose lipolysis, and hepatic gluconeogenesis\result in hyperglycaemia. This causes the compensatory release of insulin from the pancreas and hyperinsulinemia. DNL, de novo lipogenesis; DAG, diacylglycerol; FFA, free fatty acid; IR, insulin resistance; VLDL, very low-density lipoprotein.

Insulin resistance has been related to the Western diet via a variety of mechanisms [174]. These include high-glycaemic diet-related dysbiosis [54,103], chronic inflammation, and intracellular accumulation of metabolic intermediates [155]. These mechanisms may act individually or together to produce the observed features of IR. A graphical description is given in Figure 3. 

### 4.4. Evolutionary Adaptive Role of Insulin Resistance in PCOS

Human survival has relied on the ability to alter our physiology according to the changing demands of the environment or to different internal states during various life stages [175]. In evolutionary terms, physiological IR is an adaptive survival mechanism that allows organisms to selectively modulate cellular and tissue responses to a variety of environmental challenges (infection, starvation, dehydration, psychological stress, and physical stress from injury) [156,176,177] and internal states (pregnancy, puberty, and adolescence) [178,179]. Pathological IR is a detrimental condition associated with metabolic syndrome, other chronic diseases, and PCOS [173]. The suggested mechanisms and evolutionary adaptive roles of insulin resistance are discussed in the following sections.

#### 4.4.1. Insulin Resistance and Infection

Bidirectional immune-endocrine interactions regulate metabolism in the context of infection to ensure pathogen clearance, provide the appropriate metabolic requirements, and restore homeostasis [175,176]. The immune system uses PRR-activated cytokines (see Section 3.2.3) to communicate with the endocrine system and modify the responsiveness of peripheral tissues to endocrine signals. Immune cells are highly dependent on glucose for metabolism and are responsible for nearly 20% of total body energy consumption [180]. This can rise to 30% during infection and needs an effective and rapid response system to facilitate the switch from oxidative phosphorylation to more nutrient-intensive glycolytic metabolism [176]. In addition, inflammatory cytokines can influence processes normally regulated by the endocrine system, such as hunger, temperature, insulin sensitivity, and glucose uptake. Similarly, the endocrine system can regulate immune cells via a complex system of hormones (leptin, adiponectin, and insulin), receptors, nutrient sensors, and metabolic signalling pathways [176,180].

When an infectious threat is detected by the immune system, the body activates neuroendocrine components of the HPA-axis and SNS (see Section 3.3) to increase glucocorticoid and catecholamine release [180]. The HPA-axis and SNS act together with a state of cytokine-mediated IR, to mobilize free fatty acids from adipose tissue, and glucose from hepatic glycogen to ensure an appropriate supply of energy to immune cells. The ability to regulate insulin sensitivity and selectively redistribute glucose and energy, is an adaptive survival mechanism conserved in many species throughout evolutionary history [175].

In contemporary society, stress is more likely to be psychological rather than physical or infectious, but the systemic stress response is similar [175]. The HPA-axis and SNS are activated, IR is implemented, but the metabolic demand is much less, and the mobilized energy is re-stored in adipose tissue. The stress is often protracted resulting in anxiety, increased appetite from cortisol excess and leptin resistance, stress eating, and central obesity [181]. In addition, sustained adverse nutritional and environmental exposures can result in “trained immunity” (adaptive changes to innate immune responses) that may partly explain IR-related pathologies [182]. In evolutionary terms, chronic IR represents an evolutionary mismatch between ancestral survival responses and modern cultural demands and lifestyles. Women with PCOS experience increased levels of anxiety, depression, and stress, and the International Guidelines recommend screening for emotional well being [2,183].

#### 4.4.2. Insulin Resistance, Starvation and Dehydration

Insulin is an anabolic hormone that promotes cellular growth and energy storage in adipose tissue, liver, and muscle [155]. Prolonged calorie restriction during starvation activates adaptive survival mechanisms that reduce energy expenditure and prevent muscle loss [184]. Clamp studies have demonstrated that starvation-related IR is caused by decreased insulin signalling and reduced glucose uptake [185]. The implementation of IR in starvation states diminishes the oxidation of glucose, provides energy by mobilizing fatty acids from adipose tissue, limits protein loss, and redirects energy to in-demand organs [184].

Hyperinsulinemia may have played a role in preventing dehydration in ancestral populations [156]. Insulin regulates sodium channels and increases the reabsorption of sodium and water in the renal tubules [186]. Hyperinsulinemia may increase blood volume and blood pressure by this mechanism or via activation of the renin-angiotensin-aldosterone system [187]. Insulin also stimulates nitric oxide, resulting in vasodilation and decreased blood pressure. Insulin resistance results in selective impairment of the nitric oxide pathway, and hyperinsulinemia may activate the MAPK signalling pathway, cause vasoconstriction and elevated blood pressure [188]. The combined effects of these mechanisms may contribute to the maintenance of blood volume and increased cerebral perfusion during starvation. Pathological activation of these mechanisms could contribute to the increased risk of hypertension and metabolic syndrome observed in women with PCOS [189].

Women with a PCOS phenotype may have had an evolutionary advantage from having a better response to infection, dehydration, and starvation but are now more susceptible to stress, hypertension, metabolic syndrome, and PCOS as a result of contemporary lifestyle and environmental exposures [1,5].

#### 4.4.3. Insulin Resistance and Pregnancy

Insulin sensitivity decreases throughout pregnancy and is an evolutionary-conserved mechanism to limit maternal glucose use and shunt energy to the foetus, particularly during the second half of gestation [178]. Insulin sensitivity gradually decreases up to 20 weeks gestation, followed by a more rapid decrease to 50% of the non-pregnant values by 40 weeks in normal pregnancy [190]. The pancreatic beta-cells respond by increasing insulin secretion by up to 250% to maintain euglycaemia [191]. An inability to secrete adequate amounts of insulin results in elevated blood sugar levels and Gestational Diabetes Mellitis (GDM).

Maternal IR results in the use of more lipids for energy by the mother and spares carbohydrates for the foetus. The decreased maternal response to insulin is mediated by oestrogen, progesterone, human placental lactogen, human placental growth hormone, cortisol, prolactin, inflammatory cytokines, adipokines, exosomes, and the microbiome [178,192,193]. Clamp studies in late pregnancy show that hepatic gluconeogenesis is only 80% suppressed in late pregnancy in women with GDM compared to non-diabetic pregnant women [191]. Insulin sensitivity is also affected by obesity, and fat mass increases in both lean and obese women during pregnancy. Women with PCOS have higher gestational weight gain and proportionately more visceral adiposity, which is associated with greater increases in IR and GDM [178].

Women with PCOS have a 25–50% chance of developing GDM in pregnancy [194,195]. Women with GDM have up to a 50% risk of developing T2DM in the 5–10 years following pregnancy [196,197]. The population-attributable risk of PCOS to T2DM has been estimated at 19–28% [17]. Therefore, up to 28% of adult women with T2DM have pre-existing PCOS that progresses to T2DM. Up to 50% of individuals diagnosed with T2DM have complications (retinopathy, nephropathy, neuropathy, and vascular) at the time of diagnosis [198]. The International Diabetes Federation estimates that there are approximately 537 million diabetics in the world, and women with PCOS, therefore, make a significant contribution to this global epidemic [199]. These data support the characterization of PCOS as a progressive metabolic disorder. Recent studies have demonstrated that the risk of progression of GDM to T2DM can be reduced by greater than 90% by dietary and lifestyle interventions [200]. PCOS, therefore, appears to be a progressive metabolic disorder that is preventable.

In summary, the downregulation of insulin signalling and implementation of IR can be viewed as a physiological adaptive mechanism that facilitates the switch from an anabolic to a catabolic state. This results in the mobilization of fatty acids from adipose tissue, increased gluconeogenesis, release of glucose from the liver, and redistribution of energy to the immune system and brain. This process is mediated by the cooperative interaction of the immune, nervous, and endocrine systems. Chronic overactivation of any of these mechanisms creates a disturbance to whole-body homeostasis that results in chronic inflammation and IR. Women with PCOS appear to have a genetically determined proinflammatory design and increased insulin sensitivity that would be protective in an ancestral environment but becomes maladaptive in modern society.

### 4.5. Insulin Resistance and Hyperandrogenism

Androgens play an essential role in many aspects of male and female physiology, metabolism, sex differentiation, and reproductive biology [201]. Optimal health is achieved when bioactive metabolites (testosterone, dihydrotestosterone) are maintained in a defined homeostatic range. In women, low levels of androgens have been associated with endometriosis and high levels with PCOS [202]. As with all other pathophysiological processes in PCOS, there appears to be a bidirectional relationship between hyperinsulinemia and hyperandrogenism. Hyperinsulinemia can cause hyperandrogenism via mechanisms discussed below, and hyperinsulinemia has been shown to occur after the administration of androgens in clamp studies [203,204]. This may represent a self-reinforcing positive feed-forward mechanism to augment the effects of IR.

Ovarian androgen synthesis occurs in a cooperative two-step process involving luteinizing hormone (LH) stimulation of steroidogenesis in the theca cell, transfer of androstenedione to the granulosa cells, and follicle-stimulating hormone (FSH) induced production of testosterone [205]. Androgens are irreversibly converted to oestrogen in granulosa cells by the rate-limiting aromatase enzyme. Aromatase belongs to the cytochrome P450 superfamily and is the product of the CYP19A1 gene. The principal role of aromatase is to convert androgens to oestrogen. Disruption of aromatase function can lead to elevated or reduced levels of androgens or estrogen, which are associated with a wide variety of common pathologies (endometriosis, osteoporosis, hypogonadism, Alzheimer’s disease, cancer, and PCOS) [201]. Aromatase is crucial for many metabolic functions due to its role as an estrogen biosynthetic enzyme (glucose and lipid homeostasis, bone mineralization, and brain function) [206]. Ovarian aromatase activity is an important regulator of ovulation and sequential endometrial changes during the normal menstrual cycle. Dysregulation of aromatase has been linked to altered steroidogenesis, hyperandrogenism, impaired folliculogenesis, and anovulation, and is thought to play a central role in the pathogenesis of PCOS [207,208].

Structural models used to investigate the molecular evolution of aromatase have characterized the amino-acid sequences and configurations, substrate recognition sites, catalytic mechanisms, and inhibitor specificities [209,210,211]. There is a complex molecular network that regulates the normal physiology of aromatase activity, and subsequent androgen and estrogen production to maintain tissue-specific homeostasis and function [211]. The CYP19A1 gene and aromatase enzyme are present in virtually all vertebrates and have been identified in many invertebrates. The core structure, active site amino acid sequences, and substrate recognition sites have been highly conserved throughout evolutionary history [209]. In humans, there are eleven promoters that regulate tissue-specific aromatase gene expression. Post-translational modification by phosphorylation allows rapid modulation of aromatase activity compared to gene regulation. 

While FSH has been identified as the most important factor that regulates the expression of aromatase [207], other hormones such as melatonin and leptin also play an important stimulatory role [212,213]. In addition, hormonal contraceptives and endocrine-disrupting chemicals (Bisphenol A) have been shown to increase aromatase activity [214,215]. Multiple endogenous (insulin, leptin, adiponectin, cortisol, vitamin D, AGE, and inflammatory cytokines) [201] and exogenous molecules (glyphosate, phytoestrogens, resveratrol, curcumin, nicotine, alcohol, and azole agricultural antifungals) [216,217,218,219,220,221] can act as aromatase inhibitors and potentially contribute to hyperandrogenism in PCOS. In summary, aromatase appears to be a significant physiological intersection point for regulatory molecules involved in immune, metabolic, hormonal, and reproductive functions. In evolutionary terms, varying aromatase activity adjusts the homeostatic balance between oestrogen and androgens, modulating adaptive survival pathways in multiple tissues throughout the body.

As previously discussed, IR is thought to have evolved as an adaptation to environmental stressors, such as starvation, infection, and fear [1]. Varying levels of insulin sensitivity permits the redistribution of total body energy to organs of greater need, such as the brain, immune system, and foetus [175]. In addition, it has been proposed that the development of selective insulin resistance is a mechanism that activates specific behavioural and reproductive survival strategies [43]. Specific cells and tissues are protected from developing IR, including the brain, immune cells, placenta, and ovaries [222,223]. Areas of the brain do not develop IR and benefit from the redistribution of glucose from muscle and fat tissue. In pregnancy, the placenta is an insulin-independent organ, and the development of maternal IR is expected to divert more nutrients through the placenta. The ovary does not develop IR and remains sensitive to the high levels of insulin that occur in IR and hyperinsulinemia [224,225]. This may be a mechanism to downregulate fertility at times of physiological or psychological stress. In summary, both the physiological actions of insulin and the development of insulin resistance are tissue specific and may facilitate a variety of adaptive survival responses in women with PCOS.

In our modern environment, insulin resistance and hyperinsulinemia are thought to be primary factors in the development of hyperandrogenism in PCOS, in addition to chronic inflammation (discussed in Section 3.4) [225]. Insulin has several known mechanisms that can increase androgen levels in the serum, liver, and ovaries [67,225]. Insulin is reported to stimulate ovarian androgen production directly (via the PI3K and MAPK pathways) or indirectly by augmenting LH-stimulated androgen synthesis [67]. Insulin increases the availability of insulin-like growth factor and decreases its binding protein, resulting in increased androgen stimulation [226]. Insulin can increase the amplitude of gonadotropin-releasing hormone-stimulated LH pulses [227] that are known to occur in PCOS. Both insulin and testosterone decrease hepatic production of sex hormone-binding globulin, resulting in increased free testosterone [228]. In addition, hyperinsulinemia stimulates the HPA axis leading to increased adrenal androgen production [229].

Accumulating evidence suggests that the ovaries are not the primary abnormality in PCOS [225,230,231]. Women with PCOS often respond promptly to gonadotropin stimulation with clomiphene and gonadotropin-releasing hormone agonists or antiandrogenic compounds [232]. Furthermore, lifestyle interventions, such as weight loss and exercise, reduce IR and insulin levels, normalize gonadotropin secretion, and regulate menstrual cycles in women with PCOS [233]. Therapy with metformin results in reduced androgens and restores ovulation by reducing insulin levels and altering the effect of insulin on androgen biosynthesis and theca cell proliferation [234]. As discussed previously, inflammation also contributes to the pathophysiology of hyperandrogenism. Lifestyle interventions reduce both insulin levels and inflammation, highlighting the likelihood that hyperinsulinemia and chronic inflammation act together to alter ovarian steroidogenesis, increase androgen levels, impair follicular development, and reduce ovulation [10,235]. From an evolutionary perspective, inhibition of aromatase may act as another physiological mechanism (in addition to hypothalamic effects discussed in Section 3.3) to downregulate reproduction until inflammatory and metabolic stressors are contained.

## 5. Evolutionary Significance of Adipose Tissue in PCOS

Hundreds of millions of years of evolution have shaped adipose tissue (AT) into its current form [236,237,238,239]. Taking an evolutionary perspective provides insight into the complex range of AT-related adaptive survival functions that form part of the network of interdependent homeostatic systems previously discussed. Adipose tissue is involved in a variety of functions, including immune responses (innate, adaptive, and inflammatory), metabolism (glucose and lipid metabolism, appetite regulation, maintenance of body weight, and insulin resistance), and reproduction (pregnancy, lactation, and hyperandrogenism) [236,240]. Adipose tissue has a bidirectional relationship with the neuroendocrine, immune, metabolic, and reproductive systems that facilitate these functions. This communication is achieved via a variety of cellular receptors and the secretion of a large number of signalling molecules. These include adipokines (adiponectin, leptin, resistin, visfatin, retinol-binding protein 4, pigment epithelium-derived factor, endocannabinoids, and many more), cytokines (50 cytokines have been identified), metabolites, lipids, non-coding RNAs or extracellular vesicles, and chemokines [239,241].

Adipose tissue contains a diverse range of cell types, such as immune cells (macrophages, monocytes, granulocytes, and T-cells), stromal cells, fibroblasts, preadipocytes, and adipocytes [240,241] and is divided into brown AT (BAT) and white AT (WAT). WAT is classified by its anatomical location as subcutaneous AT (SAT) or visceral AT (VAT) [242,243]. Brown AT is primarily involved in thermoregulation and there are distinct differences between the location, structure, and function of VAT and SAT. 

Subcutaneous AT functions as an endocrine organ and energy storage depot and represents a normal physiological buffer to store excess consumed energy. This energy can be released during periods of limited food availability and is an important evolutionary-conserved adaptive survival mechanism [5]. Carbohydrates (glucose and fructose) that are not converted to glycogen are metabolized to triglycerides, and along with dietary lipids, are transported to AT, and stored in lipid droplets [237]. Subcutaneous AT is widely distributed but predominantly located in the abdominal wall (males) and femurogluteal region (females). The lower body distribution of SAT in women is thought to have evolved to provide insulation in the cooler temperatures of the Pleistocene open Savana [244]. This fat distribution is unique to human females and may have provided extra energy storage during lactation and pregnancy [175]. 

Genetic differences in the ability to store lipids in SAT may partly explain the apparent lean paradox in 20% of women with PCOS (see reference [1] for detailed discussion) [1]. According to the “adipose expandability hypothesis”, when the genetically determined storage capacity is exceeded, or the ability to generate new adipocytes is impaired, fat begins to accumulate in areas outside SAT [245]. In addition, excess lipid accumulation in SAT leads to adipocyte hypertrophy, hypoxia, insulin resistance, inflammation, and release of inflammatory cytokines into the circulation [246]. Chronic stress, hyperandrogenism, chronic inflammation, and insulin resistance found in women with PCOS may contribute to the accumulation of additional VAT [241,247,248].

Up to 80% of women with PCOS are overweight or obese [249]. Both lean and obese women with PCOS have been found to have an increased proportion of VAT using a variety of body composition assessment methods [250]. In addition, there are many anatomical and physiological differences between VAT and SAT. Visceral AT contains larger adipocytes, is more vascular, has more glucocorticoid, androgen, ẞ-adrenoreceptors, angiotensinogen, adiponectin and leptin, has less oestrogen receptors, and reduced fatty acid uptake [239,240,241]. Visceral AT also has increased catecholamine-induced lipolysis, greater insulin sensitivity, and produces more inflammatory cytokines [236,240]. It has been proposed that having an increased proportion of VAT provides an adaptive survival advantage against infection [236]. This has been called the “VAT prioritization hypothesis” and is supported by multiple lines of evidence.

Most VAT is in the omentum and mesentery [236]. Mesenteric VAT surrounds the small intestine and is the next line of defence against pathogens and endotoxins (LPS) translocated from the intestine, following passage through the submucosal immune system [251]. The mesenteric VAT contains large numbers of immune cells that can mount a rapid energy-intensive immune response in case of infection. Visceral adipocytes, such as lymph nodes, are specialized to store diet-derived polyunsaturated fatty acids that are required for an immune response. Mesenteric VAT contains lymphoid clusters that expand in response to inflammation and infection [251]. Blood from the mesenteric and omental VAT drains into the portal vein and is moved directly to the liver. The junction of the portal vein and liver contains another cluster of immunologically important cells, stellate cells (specialized fat cells), and Kupffer cells (macrophages) that provide a further layer of immunological protection [252]. The VAT prioritization hypothesis proposes that these evolutionary adaptive changes are developmentally programmed in a similar way to the proposed developmental programming of PCOS. Readers are referred to articles by West-Eberhard [236] and Parker [253] for detailed discussion.

In summary, adipose tissue and obesity have a significant bidirectional role in the pathophysiology of chronic systemic inflammation and insulin resistance in women with PCOS. The anatomical and functional redistribution of adipose tissue in women with PCOS may have adaptive survival benefits in an ancestral environment (improved energy storage capacity and greater protection from infection) that become maladaptive in response to contemporary lifestyle and environmental exposures (diet, inactivity, and stress). In addition, adipose tissue is closely linked to the reproductive system and clearly plays a significant role in the pathophysiology of PCOS.

## 6. Central Role of the Microbiome in the Pathogenesis of PCOS

The microbiota is the sum of microbial organisms (bacteria, viruses, archaea, and fungi) that inhabit the interface between the external environment or habitat and the internal environment of the human body [254]. This interface mainly occurs at mucosal surfaces (nasal, oral, respiratory, gastrointestinal, and genitourinary), eyes, and skin. The gastrointestinal (GI) microbiome (microbial organisms and their genetic material) makes up 80% of the microbiota and is now considered to have a central role in human health and disease, including PCOS [103,255,256]. The functions of the microbiome include inhibition of pathogen colonization, regulation of the mucosal and systemic immune systems, alteration of metabolism, energy balance, hormonal action, maintenance of the integrity of the GI barrier, and bidirectional signalling with most organs and tissues throughout the body (gut-brain, gut-bone, gut-immune, gut-liver, etc). The GI microbiome, therefore, forms part of the whole-body homeostatic regulatory framework that maintains health and is now appreciated to be part of the human-microbe meta-organism [44].

Microbiome eubiosis (a balanced microbial ecosystem) can be disrupted by external factors (lifestyle, diet, environmental chemicals, pathogenic microbes, and medications), or endogenous factors (hormones, circadian, exercise, and stress). Many of these factors can cause dysbiosis (an imbalance of the composition, metabolism, or distribution of the microbiome associated with a negative health outcome) and have been investigated for their role in a wide range of human diseases [257]. These include obesity, diabetes, gestational diabetes, metabolic syndrome, cardiovascular disease, inflammatory bowel disease, multiple sclerosis, dementia, PCOS, and many others [258,259]. Lifestyle-related disturbance to the balanced microbial ecosystem contributes to a variety of diseases, depending on the level of exposure, length of exposure, combination of exposures, and individual disease susceptibility [257]. Accumulating evidence suggests that dysbiosis plays a fundamental role in the pathogenesis of PCOS [54,103].

The dysbiosis of gut microbiota theory of PCOS proposes that poor-quality Western-style diets (high-glycaemic, high fat, high calorie, highly processed, low nutrient, low fibre), result in an imbalanced microbiome which induces increased GI permeability and results in endotoxin-mediated chronic inflammation [54]. Dysbiosis results in breakdown of the GI barrier function (loss of protective mucous, activation of the zonulin pathway, and breakdown of intercellular tight junctions), and release of lipopolysaccharide from the cell walls of gram-negative bacteria which can traverse the “leaky gut.” Lipopolysaccharide binds with lipopolysaccharide binding protein which together bind with Toll-like receptor 4 on the surface of submucosal macrophages. This activates the NF-κB inflammatory pathway, resulting in release of inflammatory cytokines. Continuing ingestion of a poor-quality diet results in chronic inflammation, insulin resistance, hyperandrogenism, ovulatory dysfunction, and the clinical features of PCOS [1,54]. 

A recent review of 31 proof-of-concept studies that specifically investigated this mechanism, concluded that preliminary evidence supports this theory [103]. Most studies showed reduced alpha-diversity (lower numbers of bacterial taxa) of the microbiome in women with PCOS compared to healthy individuals. Despite this finding, no specific microbial signature has been found in women with PCOS [255]. This is likely due to immense individual variability in microbiomes, the existence of functional redundancy (microbiotas contain many species and strains that can perform the same function) [260], as well as the fact that PCOS is a quantitative trait (interaction between environment and individual). Individual variation in the composition of the microbiome has likely contributed to the ability of humans to adapt to so many varied environments and types of diets, throughout evolutionary history [254]. 

Many other dysbiosis-related pathophysiological mechanisms have been proposed and investigated since the dysbiosis model was proposed in 2012. These include altered choline pathways, changes in bile acid metabolites that affect inflammation, glucose and lipid metabolism, processes involved in energy absorption, short-chain fatty acids, effects on gastrointestinal hormones, and other host metabolites (trimethylamine N-oxide, lactate, primary bile acids) [255,261]. Many food components are not digested in the small intestine and transit to the colon where they are fermented by the colonic microbiota. Bacterial conversion of these compounds results in a wide variety of metabolites that enter the host, alter immunity, metabolism, hormones and reproduction, and can influence the risk of disease [77]. Fermentation of undigested carbohydrates (soluble fibre), proteins and plant-derived phytochemicals (polyphenols) results in a range of bioactive metabolites, such as short-chain fatty acids, amines, secondary metabolites of polyphenols, and gases (hydrogen, hydrogen sulphide, and methane). It is likely that future research will identify a range of pathophysiological mechanisms related to the role of dysbiosis in the pathogenesis of PCOS.

In summary, as with the rest of the biological components involved in PCOS, the microbiome appears to have a significant bidirectional role in the pathophysiology of chronic systemic inflammation, insulin resistance, hyperandrogenism, and ovulatory dysfunction. Dysbiosis is an important part of the evolutionary model and represents a maladaptive response of the microbiome to contemporary lifestyle and the environment. Dysbiosis is a modifiable change that can be reversed with lifestyle, diet, prebiotics, and probiotics [103]. 

## 7. Environmental and Endocrine Disrupting Chemicals in PCOS

Endocrine-disrupting chemicals are a global problem for human health and the environment [262]. There is no doubt that anthropomorphic chemicals interfere with human physiology and have adverse health effects [263,264,265,266]. Human exposure mainly occurs through mucosal surfaces (oral, gastrointestinal, respiratory, and genitourinary) or via dermal absorption. EDC exposure is ubiquitous and numerous international organizations have issued warnings to doctors and patients, regarding the possible dangers to human health and pregnant women. These include The Royal College of Obstetricians and Gynaecologists [264], the International Federation of Gynecology and Obstetrics [265], and the Endocrine Society [266]. They have recommended that all pregnant women be advised of the possible risks of EDC, and that education programmes be developed to inform health professionals of the risks.

Endocrine disrupting chemicals have a significant role in chronic systemic inflammation [267], insulin resistance [268], hyperandrogenism [269], microbiome disruption [270] neuroendocrine imbalance [271], obesity [272], oxidative stress [273], AGE [274], and inflammasomes [275], all of which are involved in the pathophysiology of PCOS. Intervention trials, randomized-controlled trials (RCT), and systematic reviews of RCT will never be possible or ethical in humans. Hypotheses about mechanisms of causation need to be based on observational studies in humans, human bio-monitoring studies, intervention studies in animals, and disasters from inadvertent human exposure [262]. Large amounts of data are already available that provide “evidence” of the potential harmful effects of EDC in humans [267,268,269,270,271,272,273,274,275,276]. The implementation of the “Precautionary Principle” [277] is supported by a long list of previous known disasters that have occurred due to delayed action or negligent inaction (diethylstilboestrol, thalidomide, nicotine, dioxin, asbestos, and mesh) [278].

Many EDC has been investigated and implicated in the pathogenesis and pathophysiology of PCOS. These include heavy metals, persistent organic pollutants, polychlorinated biphenyls, organochloride pesticides, air pollutants, pesticides, herbicides, nanomaterials, and plastics [271,279,280,281,282,283]. EDC can act individually, but humans are usually exposed to mixtures of chemicals on a regular basis [284]. EDC has been identified in most body fluids in women with PCOS [285,286]. Six classes of EDC have been shown to cross the placenta [285,287], and EDC may be involved in transgenerational transmission of PCOS [103,288]. The anthropometric production of EDC is intimately connected to climate change and related adverse health outcomes, and climate change can exacerbate the effects of EDC [284]. Improved regulatory processes, bio-detection methods, climate change mitigation strategies, and dietary interventions, can result in reduced exposure to EDC and other environmental chemicals [289].

Women with a genetic susceptibility to PCOS may be at increased risk of adverse effects of EDC due to having a heightened proinflammatory design (Section 3.1) and increased metabolic sensitivity (Section 4.2). In addition, the in-utero developmental effects of EDC can be inherited by transgenerational transmission [253,278]. Every effort should be made to inform women with PCOS about the potential risks of environmental chemicals and discuss ways to avoid or minimize exposure. A detailed discussion of the mechanisms of action of EDC, animal, and epidemiological studies related to PCOS is beyond the scope of this review. Readers are referred to published reports for further information [280,281,282,290]. 

## 8. Evolutionary Model of PCOS and the Hallmarks of Health

There has been a gradual paradigm shift in conceptualizing the causes of health and disease over the past 20 years. Seminal publications on the “Hallmarks of Cancer” summarize the properties of malignant cells and their interaction with their non-malignant environment [291]. The “Hallmarks of Aging” focus on the interactions of molecular, cellular, and systemic processes that explain the deterioration of organisms over time [292]. This is a paradigm shift that signals a move away from conventional, anatomical, and physiological-based conceptions of disease (individual cells, tissues, organs, and systems) to a more “organizational” structure focused on factors that are causatively involved in maintaining homeostasis and equilibrium.

The “Hallmarks of Health” has endeavoured to define health as a “compendium of organizational and dynamic features that maintain physiology [44].” This contrasts with the usual definition of health as the “absence of disease.” The current conception of PCOS as a polygenic disorder (collection of normal alleles) that is programmed in-utero and then manifests in adolescence following exposure to lifestyle, nutritional, and environmental influences, seems to be an excellent example of this new paradigm [1]. Many of the inter-related components of the hallmarks of health are dysregulated in women with PCOS. There are disturbances at the molecular (ROS, AGE, and metabolites), organelle (mitochondria and endoplasmic reticulum), cellular (immune, endocrine, and neural), supracellular (gastrointestinal mucosa and mucosal immunity), organ (ovary and pancreas), systemic (endocrine, reproductive, and immune), and meta-organism levels (host–microbiota interaction) (Table 1). The previous sections of this manuscript have attempted to describe some of the detailed interactions that disrupt the organizational dynamics of the hallmarks of health in women with PCOS.

The Evolutionary Model of PCOS is consistent with the hallmarks of health paradigm [1,5]. Seen from this new perspective, PCOS is a progressive disturbance of the overall organism involving multiple levels that usually operate to maintain internal homeostasis and equilibrium with the environment. Multiple contemporary lifestyle factors can derail the overall organization of the system, resulting in complex pathophysiological interactions and feedback mechanisms, that have no apparent beginning or end. All the components are inter-related and interdependent, in a system where multiple lifestyle and environmental “causative” factors operate simultaneously and synergistically. Defining PCOS in a positive way, with an evolutionary and hallmarks of health perspective, opens new opportunities for understanding this complex condition, improving patient communication and compliance, and informing future research, prevention, and intervention strategies.

## 9. Conclusions

Polycystic ovary syndrome is a common condition that affects women at all stages of the life cycle. The pathogenesis is related to a combination of nutritional and environmental exposures in a genetically susceptible individual. Polycystic ovary syndrome can be characterized as an evolutionary mismatch disorder resulting from a disturbance to the hallmarks of health. Chronic systemic inflammation and insulin resistance are core mechanisms that operate at an organismal level in the physiology of survival and play a central role in the pathophysiology of PCOS. Polycystic ovary syndrome is usually diagnosed in adolescence by the combination of oligomenorrhoea and hyperandrogenism and is a progressive condition that is associated with significant metabolic, hormonal, reproductive, and psychological problems. The symptoms and chronic sequelae can be controlled and prevented by lifestyle interventions, and the diagnosis of PCOS provides the ideal opportunity for the prevention of chronic disease. This narrative review has outlined some components of the complex web of biological and pathophysiological changes that contribute to the pathogenesis of PCOS.

## Figures and Tables

**Table 1:** Dysregulation of the Hallmarks of Health in Polycystic Ovary Syndrome.

Hallmark	Polycystic Ovary Syndrome
Barrier integrity	Gastrointestinal permeability Respiratory mucosa
Containment of local perturbations	Mucosal and adaptive immunity ROS, AGE, DAMPS, PAMPS
Recycling and turnover	Autophagy, apoptosis, pyroptosis GI stem cell turnover
Integration of circuitries	Metabolic, reproductive neurological, circadian
Rhythmic oscillations	Menstrual cycle, circadian GI peristalsis
Homeostatic resilience	Endocrine, ANS metabolic, immune, microbiome
Hormetic regulation	oxidative stress, inactivity xenohormesis ^#^
Repair and regeneration	Endoplasmic reticular stress mitochondrial stress

^#^ xenohormesis = dietary phytochemical-induced; ANS = autonomic nervous system; DAMPS = danger-associated molecular patterns; ROS = reactive oxygen species; PAMPS = pathogen-associated molecular patterns; GI = Gastrointestinal; AGE = advanced glycation end-products.

## Abbreviations

ACTH	Adrenocorticotropic Hormone
AGE	Advanced Glycation End-Products
ANS	Autonomic Nervous System
AT	Adipose Tissue
BAT	Brown Adipose Tissue
CDR	Cell Danger Response
DAMPS	Danger Associated Molecular Patterns
DNA	Deoxyribose Nucleic Acid
EDC	Endocrine Disrupting Chemical
GDM	Gestational Diabetes
GI	Gastrointestinal
GLUT	Glucose Transporter
HPA	Hypothalamic-Pituitary-Adrenal
IR	Insulin Resistance
LH	Luteinizing Hormone
LPS	Lipopolysaccharide
PAMPS	Pathogen Associated Molecular Patterns
PCOS	Polycystic Ovary Syndrome
PNS	Peripheral Nervous System
PRR	Pattern Recognition Receptors
RAGE	Receptor of Advanced Glycation End-Products
RNA	Ribose Nucleic Acid
RNS	Reactive Nitrogen Species
ROS	Reactive Oxygen Species
SAT	Subcutaneous Adipose Tissue
SNS	Sympathetic Nervous System
TLR	Toll-like Receptor
TNF	Tumour Necrosis Factor
T2DM	Type 2 Diabetes Mellitis
WAT	White Adipose Tissue
VAT	Visceral Adipose Tissue

## References

[1] Parker J., O’brien C., Hawrelak J., Gersh F.L. (2022). Polycystic Ovary Syndrome: An Evolutionary Adaptation to Lifestyle and the Environment. Int. J. Environ. Res. Public Health.

[2] Teede H.J., Misso M.L., Costello M.F., Dokras A., Laven J., Moran L., Piltonen T., Norman R.J., Andersen M., Azziz R. (2018). Recommendations from the international evidence-based guideline for the assessment and management of polycystic ovary syndrome. Fertil. Steril..

[3] Cowan S., Lim S., Alycia C., Pirotta S., Thomson R., Gibson-Helm M., Blackmore R., Naderpoor N., Bennett C., Ee C. (2023). Lifestyle management in polycystic ovary syndrome—Beyond diet and physical activity. BMC Endocr. Disord..

[4] Pei Z., Lu W., Feng Y., Xu C., Hsueh A.J.W. (2022). Out of step societal and Darwinian adaptation during evolution is the cause of multiple women’s health issues. Hum. Reprod..

[5] Dumesic D.A., Padmanabhan V., Chazenbalk G.D., Abbott D.H. (2022). Polycystic ovary syndrome as a plausible evolutionary outcome of metabolic adaptation. Reprod. Biol. Endocrinol..

[6] Pathak G., Nichter M. (2015). Polycystic ovary syndrome in globalizing India: An ecosocial perspective on an emerging lifestyle disease. Soc. Sci. Med..

[7] Parker J., O’Brien C. (2021). Evolutionary and genetic antecedents to the pathogenesis of polycystic ovary syndrome (PCOS). J. ACNEM.

[8] Aboeldalyl S., James C., Seyam E., Ibrahim E.M., Shawki H.E.D., Amer S. (2021). The role of chronic inflammation in polycystic ovarian syndrome—A systematic review and meta-analysis. Int. J. Mol. Sci..

[9] Giampaolino P., Foreste V., Di Filippo C., Gallo A., Mercorio A., Serafino P., Improda F.P., Verrazzo P., Zara G., Buonfantino C. (2021). Microbiome and PCOS: State-of-art and future aspects. Int. J. Mol. Sci..

[10] Wang J., Wu D., Guo H., Li M. (2019). Hyperandrogenemia and insulin resistance: The chief culprit of polycystic ovary syndrome. Life Sci..

[11] Palomba S., De Wilde M.A., Falbo A., Koster M.P.H., La Sala G.B., Fauser B.C.J.M. (2015). Pregnancy complications in women with polycystic ovary syndrome. Hum. Reprod. Update.

[12] Brutocao C., Zaiem F., Alsawas M., Morrow A.S., Murad M.H., Javed A. (2018). Psychiatric disorders in women with polycystic ovary syndrome: A systematic review and meta-analysis. Endocrine.

[13] Zore T., Joshi N.V., Lizneva D., Azziz R. (2017). Polycystic Ovarian Syndrome: Long-Term Health Consequences. Semin. Reprod. Med..

[14] Wu J., Yao X.Y., Shi R.X., Liu S.F., Wang X.Y. (2018). A potential link between polycystic ovary syndrome and non-alcoholic fatty liver disease: An update meta-analysis. Reprod. Health.

[15] Yumiceba V., Lopez-Cortes A., Perez-Villa A. (2020). Oncology and Pharmacogenomics Insights in Polycystic Ovary Syndrome: An Integrative Analysis. Front. Endocrinol..

[16] Du Y., Li F., Li S., Ding L., Liu M. (2023). Causal relationship between polycystic ovary syndrome and chronic kidney disease: A Mendelian randomization study. Front. Endocrinol..

[17] Rodgers R.J., Avery J.C., Moore V.M., Davies M.J., Azziz R., Stener-Victorin E., Moran L.J., Robertson S.A., Stepto N.K., Norman R.J. (2019). Complex diseases and co-morbidities: Polycystic ovary syndrome and type 2 diabetes mellitus. Endocr. Connect..

[18] Parker J. (2018). NEM: A New Paradigm for Understanding the Common Origins of the Chronic Disease Epidemic. ACNEM J..

[19] Dunaif A. (2016). Perspectives in polycystic ovary syndrome: From hair to eternity. J. Clin. Endocrinol. Metab..

[20] Day F., Karaderi T., Jones M.R., Meun C., He C., Drong A., Kraft P., Lin N., Huang H., Broer L. (2018). Large-scale genome-wide meta-analysis of polycystic ovary syndrome suggests shared genetic architecture for different diagnosis criteria. PLoS Genet..

[21] Vink J.M., Sadrzadeh S., Lambalk C.B., Boomsma D.I. (2006). Heritability of polycystic ovary syndrome in a Dutch twin-family study. J. Clin. Endocrinol. Metab..

[22] Kahsar-Miller M.D., Nixon C., Boots L.R., Go R.C., Azziz R. (2001). Prevalence of polycystic ovary syndrome (PCOS) in first-degree relatives of patients with PCOS. Fertil. Steril..

[23] Sun Q., Gao Y., Yang J., Lu J., Feng W., Yang W. (2021). Mendelian Randomization Analysis Identified Potential Genes Pleiotropically Associated with Polycystic Ovary Syndrome. Reprod. Sci..

[24] Zhu T., Goodarzi M. (2021). Causes and consequences of polycystic ovary syndrome: Insights from Mendelian Randomization. J. Clin. Endocrinol. Metab..

[25] Lyle S.M., Ahmed S., Elliott J.E., Stener-Victorin E., Nachtigal M.W., Drögemöller B.I. (2023). Transcriptome-wide association analyses identify an association between ARL14EP and polycystic ovary syndrome. J. Hum. Genet..

[26] Charifson M.A., Trumble B.C. (2019). Evolutionary origins of polycystic ovary syndrome: An environmental mismatch disorder. Evol. Med. Public Health.

[27] Shaw L.M.A., Elton S. (2008). Polycystic ovary syndrome: A transgenerational evolutionary adaptation. BJOG An Int. J. Obstet. Gynaecol..

[28] Diamanti-Kandarakis E., Piperi C. (2005). Genetics of polycystic ovary syndrome: Searching for the way out of the labyrinth. Hum. Reprod. Update.

[29] Khan M.J., Ullah A., Basit S. (2019). Genetic basis of polycystic ovary syndrome (PCOS): Current perspectives. Appl. Clin. Genet..

[30] Chodasewicz K. (2014). Evolution, reproduction and definition of life. Theory Biosci..

[31] Corbett S., Morin-Papunen L. (2013). The Polycystic Ovary Syndrome and recent human evolution. Mol. Cell. Endocrinol..

[32] Benton M.L. (2021). The influence of evolutionary history on human health and disease. Nat. Rev. Genet..

[33] Brady S.P., Bolnick D.I., Barrett R.D.H., Chapman L., Crispo E., Derry A.M., Eckert C.G., Fraser D.J., Fussmann G.F., Gonzalez A. (2019). Understanding maladaptation by uniting ecological and evolutionary perspectives. Am. Nat..

[34] Parker J. (2020). Glucose metabolism, energy production and regulation of cellular and whole-body metabolism. ACNEM J..

[35] Chantranupong L., Wolfson R.L., Sabatini D.M. (2015). Nutrient-sensing mechanisms across evolution. Cell.

[36] Holly J.M.P., Biernacka K., Perks C.M. (2019). Systemic metabolism, its regulators, and cancer: Past mistakes and future potential. Front. Endocrinol..

[37] Norman R., Teede H. (2018). A new evidence-based guideline for assessment and management of polycystic ovary syndrome. Med. J. Aust..

[38] Casarini L., Simoni M., Brigante G. (2016). Is polycystic ovary syndrome a sexual conflict? A review. Reprod. Biomed. Online.

[39] Parker J. (2016). Emerging Concepts in the Pathogenesis and Treatment of Polycystic Ovary Syndrome. Curr. Womens. Health Rev..

[40] Azziz R., Dumesic D.A., Goodarzi M.O. (2011). Polycystic ovary syndrome: An ancient disorder?. Fertil. Steril..

[41] Gluckman P.D., Low F.M., Hanson M.A. (2020). Anthropocene-related disease. Evol. Med. Public Health.

[42] Rubio-Ruiz M.E., Peredo-Escárcega A.E., Cano-Martínez A., Guarner-Lans V. (2015). An Evolutionary Perspective of Nutrition and Inflammation as Mechanisms of Cardiovascular Disease. Int. J. Evol. Biol..

[43] Watve M.G., Yajnik C.S. (2007). Evolutionary origins of insulin resistance: A behavioral switch hypothesis. BMC Evol. Biol..

[44] López-Otín C., Kroemer G. (2021). Hallmarks of Health. Cell.

[45] Ling C., Rönn T. (2019). Epigenetics in Human Obesity and Type 2 Diabetes. Cell Metab..

[46] Szukiewicz D., Trojanowski S., Kociszewska A., Szewczyk G. (2022). Modulation of the Inflammatory Response in Polycystic Ovary Syndrome (PCOS)—Searching for Epigenetic Factors. Int. J. Mol. Sci..

[47] Shorakae S., Ranasinha S., Abell S., Lambert G., Lambert E., de Courten B., Teede H. (2018). Inter-related effects of insulin resistance, hyperandrogenism, sympathetic dysfunction and chronic inflammation in PCOS. Clin. Endocrinol..

[48] Barlampa D., Bompoula M.S., Bargiota A., Kalantaridou S., Mastorakos G., Valsamakis G. (2021). Hypothalamic inflammation as a potential pathophysiologic basis for the heterogeneity of clinical, hormonal, and metabolic presentation in pcos. Nutrients.

[49] Okin D., Medzhitov R. (2012). Evolution of inflammatory diseases. Curr. Biol..

[50] Furman D., Campisi J., Verdin E., Carrera-Bastos P., Targ S., Franceschi C., Ferrucci L., Gilroy D.W., Fasano A., Miller G.W. (2019). Chronic inflammation in the etiology of disease across the life span. Nat. Med..

[51] Naviaux R.K. (2019). Metabolic features and regulation of the healing cycle—A new model for chronic disease pathogenesis and treatment. Mitochondrion.

[52] Yu S.Y., Li X.L. (2021). Pyroptosis and inflammasomes in obstetrical and gynecological diseases. Gynecol. Endocrinol..

[53] Katayama P.L., Leirão I.P., Kanashiro A., Luiz J.P.M., Cunha F.Q., Navegantes L.C.C., Menani J.V., Zoccal D.B., Colombari D.S.A., Colombari E. (2022). The carotid body detects circulating tumor necrosis factor-alpha to activate a sympathetic anti-inflammatory reflex. Brain Behav. Immun..

[54] Tremellen K., Pearce K. (2012). Dysbiosis of Gut Microbiota (DOGMA)—A novel theory for the development of polycystic ovarian syndrome. Med. Hypotheses.

[55] Xiong P., Zhang F., Liu F., Zhao J., Huang X. (2023). Metaflammation in glucolipid metabolic disorders: Pathogenesis and treatment. Biomed. Pharmacother..

[56] Netea M.G., Balkwill F., Chonchol M., Cominelli F., Donath M.Y., Giamarellos-Bourboulis E.J., Golenbock D., Gresnigt M.S., Heneka M.T., Hoffman H.M. (2017). A guiding map for inflammation. Nat. Immunol..

[57] Zhang H., Zhu J., Gong Z., Zhu J.K. (2022). Abiotic stress responses in plants. Nat. Rev. Genet..

[58] Khan R.N., Hay D.P. (2015). A clear and present danger: Inflammasomes DAMPing down disorders of pregnancy. Hum. Reprod. Update.

[59] Amarante-Mendes G.P., Adjemian S., Branco L.M., Zanetti L.C., Weinlich R., Bortoluci K.R. (2018). Pattern recognition receptors and the host cell death molecular machinery. Front. Immunol..

[60] Cavalier-Smith T. (2006). Cell evolution and Earth history: Stasis and revolution. Philos. Trans. R. Soc. B Biol. Sci..

[61] Tang K.H., Blankenship R.E. (2010). Both forward and reverse TCA cycles operate in green sulfur bacteria. J. Biol. Chem..

[62] Naviaux R.K. (2012). Oxidative shielding or oxidative stress?. J. Pharmacol. Exp. Ther..

[63] Galluzzi L., Vitale I., Aaronson S.A., Abrams J.M., Adam D., Agostinis P., Alnemri E.S., Altucci L., Amelio I., Andrews D.W. (2018). Molecular mechanisms of cell death: Recommendations of the Nomenclature Committee on Cell Death 2018. Cell Death Differ..

[64] Dabravolski S.A., Nikiforov N.G., Eid A.H., Nedosugova L.V., Starodubova A.V., Popkova T.V., Bezsonov E.E., Orekhov A.N. (2021). Mitochondrial dysfunction and chronic inflammation in polycystic ovary syndrome. Int. J. Mol. Sci..

[65] Zhao H., Zhao Y., Li T., Li M., Li J., Li R., Liu P., Yu Y., Qiao J. (2015). Metabolism alteration in follicular niche: The nexus among intermediary metabolism, mitochondrial function, and classic polycystic ovary syndrome. Free Radic. Biol. Med..

[66] Mohammadi M. (2017). Oxidative Stress and Polycystic Ovary Syndrome: A Brief Review. Int. J. Prev. Med..

[67] Zuo T., Zhu M., Xu W. (2016). Roles of oxidative stress in polycystic ovary syndrome and cancers. Oxid. Med. Cell. Longev..

[68] Sharifi-Rad M., Anil Kumar N.V., Zucca P., Varoni E.M., Dini L., Panzarini E., Rajkovic J., Tsouh Fokou P.V., Azzini E., Peluso I. (2020). Lifestyle, Oxidative Stress, and Antioxidants: Back and Forth in the Pathophysiology of Chronic Diseases. Front. Physiol..

[69] Herman R., Jensterle M., Janež A., Goričar K., Dolžan V. (2020). Genetic variability in antioxidative and inflammatory pathways modifies the risk for PCOs and influences metabolic profile of the syndrome. Metabolites.

[70] Diamanti-Kandarakis E., Dunaif A. (2012). Insulin resistance and the polycystic ovary syndrome revisited: An update on mechanisms and implications. Endocr. Rev..

[71] Rashidi B., Haghollahi F., Shariat M., Zayerii F. (2009). The Effects of Calcium-Vitamin D and Metformin on Polycystic Ovary Syndrome: A Pilot Study. Taiwan. J. Obstet. Gynecol..

[72] Kaya C., Erkan A.F., Cengiz S.D., Dünder I., Demirel Ö.E., Bilgihan A. (2009). Advanced oxidation protein products are increased in women with polycystic ovary syndrome: Relationship with traditional and nontraditional cardiovascular risk factors in patients with polycystic ovary syndrome. Fertil. Steril..

[73] Enechukwu C.I., Onuegbu A.J., Olisekodiaka M.J., Eleje G.U., Ikechebelu J.I., Ugboaja J.O., Amah U.K., Okwara J.E., Igwegbe A.O. (2019). Oxidative stress markers and lipid profiles of patients with polycystic ovary syndrome in a Nigerian tertiary hospital. Obstet. Gynecol. Sci..

[74] Dincer Y., Akcay T., Erdem T., Ilker Saygili E., Gundogdu S. (2005). DNA damage, DNA susceptibility to oxidation and glutathione level in women with polycystic ovary syndrome. Scand. J. Clin. Lab. Investig..

[75] Noormohammadi M., Eslamian G., Malek S., Shoaibinobarian N., Mirmohammadali S.N. (2022). The association between fertility diet score and polycystic ovary syndrome: A Case-Control study. Health Care Women Int..

[76] Shoaibinobarian N., Eslamian G., Noormohammadi M., Malek S., Rouhani S., Mirmohammadali S.N. (2022). Dietary Total Antioxidant Capacity and Risk of Polycystic Ovary Syndrome: A Case-Control Study. Int. J. Fertil. Steril..

[77] Parker J., Hawrelak J., Gersh F. (2021). Nutritional Role of Polyphenols as a Component of a Wholefood Diet in the Management of Polycystic Ovary Syndrome. Australas. Coll. Nutr. Environ. Med. J..

[78] Piperi C., Adamopoulos C., Dalagiorgou G., Diamanti-Kandarakis E., Papavassiliou A.G. (2012). Crosstalk between advanced glycation and endoplasmic reticulum stress: Emerging therapeutic targeting for metabolic diseases. J. Clin. Endocrinol. Metab..

[79] Inagi R. (2011). Inhibitors of advanced glycation and endoplasmic reticulum stress. Methods in Enzymology.

[80] Goldberg T., Cai W., Peppa M., Dardaine V., Baliga B.S., Uribarri J., Vlassara H. (2004). Advanced glycoxidation end products in commonly consumed foods. J. Am. Diet. Assoc..

[81] Garg D., Merhi Z. (2015). Advanced glycation end products: Link between diet and ovulatory dysfunction in PCOS?. Nutrients.

[82] Basta G. (2008). Receptor for advanced glycation endproducts and atherosclerosis: From basic mechanisms to clinical implications. Atherosclerosis.

[83] Kathryn C., Tan B., Sammy W., Shiu M., Wong Y. (2011). Xystus Tam Serum advanced glycation end products (AGEs) are associated with insulin resistance. Diabetes. Metab. Res. Rev..

[84] Palimeri S., Palioura E., Diamanti-Kandarakis E. (2015). Current perspectives on the health risks associated with the consumption of advanced glycation end products: Recommendations for dietary management. Diabetes Metab. Syndr. Obes. Targets Ther..

[85] Diamanti-Kandarakis E., Piperi C., Patsouris E., Korkolopoulou P., Panidis D., Pawelczyk L., Papavassiliou A.G., Duleba A.J. (2007). Immunohistochemical localization of advanced glycation end-products (AGEs) and their receptor (RAGE) in polycystic and normal ovaries. Histochem. Cell Biol..

[86] Azhary J.M.K., Harada M., Kunitomi C., Kusamoto A., Takahashi N., Nose E., Oi N., Wada-Hiraike O., Urata Y., Hirata T. (2020). Androgens Increase Accumulation of Advanced Glycation End Products in Granulosa Cells by Activating ER Stress in PCOS. Endocrinology.

[87] Tatone C., Di Emidio G., Placidi M., Rossi G., Ruggieri S., Taccaliti C., D’Alfonso A., Amicarelli F., Guido M. (2021). AGEs-related dysfunctions in PCOS: Evidence from animal and clinical research. J. Endocrinol..

[88] Garg D., Merhi Z. (2016). Relationship between Advanced Glycation End Products and Steroidogenesis in PCOS. Reprod. Biol. Endocrinol..

[89] Van Der Lugt T., Weseler A.R., Gebbink W.A., Vrolijk M.F., Opperhuizen A., Bast A. (2018). Dietary advanced glycation endproducts induce an inflammatory response in human macrophages in vitro. Nutrients.

[90] Uribarri J., del Castillo M.D., de la Maza M.P., Filip R., Gugliucci A., Luevano-Contreras C., Macías-Cervantes M.H., Markowicz Bastos D.H., Medrano A., Menini T. (2015). Dietary advanced glycation end products and their role in health and disease. Adv. Nutr..

[91] Tantalaki E., Piperi C., Livadas S., Kollias A., Adamopoulos C., Koulouri A., Christakou C., Diamanti-Kandarakis E. (2014). Impact of dietary modification of advanced glycation end products (AGEs) on the hormonal and metabolic profile of women with polycystic ovary syndrome (PCOS). Hormones.

[92] Vlassara H., Striker G.E. (2011). AGE restriction in diabetes mellitus: A paradigm shift. Nat. Rev. Endocrinol..

[93] Janeway C.A. (1989). Approaching the asymptote? Evolution and revolution in immunology. Cold Spring Harb. Symp. Quant. Biol..

[94] Matzinger P. (1994). Tolerance, danger, and the extended family. Annu. Rev. Immunol..

[95] Naviaux R.K. (2014). Metabolic features of the cell danger response. Mitochondrion.

[96] Pakos-Zebrucka K., Koryga I., Mnich K., Ljujic M., Samali A., Gorman A.M. (2016). The integrated stress response. EMBO Rep..

[97] Costa-Mattioli M., Walter P. (2020). The integrated stress response: From mechanism to disease. Science.

[98] Li D., Wu M. (2021). Pattern recognition receptors in health and diseases. Signal Transduct. Target. Ther..

[99] Moretti J., Blander J.M. (2014). Insights into phagocytosis-coupled activation of pattern recognition receptors and inflammasomes. Curr. Opin. Immunol..

[100] Zhou F., Li C., Zhang S.Y. (2020). NLRP3 inflammasome: A new therapeutic target for high-risk reproductive disorders?. Chin. Med. J..

[101] Moretti J., Blander J.M. (2017). Cell-autonomous stress responses in innate immunity. J. Leukoc. Biol..

[102] Cani P.D., Amar J., Iglesias M.A., Poggi M., Knauf C., Bastelica D., Neyrinck A.M., Fava F., Tuohy K.M., Chabo C. (2007). Metabolic endotoxemia initiates obesity and insulin resistance. Diabetes.

[103] Parker J., O’Brien C., Hawrelak J. (2021). A narrative review of the role of gastrointestinal dysbiosis in the pathogenesis of polycystic ovary syndrome. Obstet. Gynecol. Sci..

[104] Murthi P., Pinar A.A., Dimitriadis E., Samuel C.S. (2020). Inflammasomes—A molecular link for altered immunoregulation and inflammation mediated vascular dysfunction in preeclampsia. Int. J. Mol. Sci..

[105] Rostamtabar M., Esmaeilzadeh M., Tourani M., Rahmani A., Baee M., Shirafkan F., Saleki K., Mirzababayi S., Ebrahimpour S., Nouri H. (2020). Pathophysiological roles of chronic low-grade inflammation in polycystic ovary syndrome. J. Cell. Physiol..

[106] Eltzschig H.K., Sitkovsky M.V., Robson S. (2013). Purinergic Signaling during Inflammation. N. Engl. J. Med..

[107] Rostamtabar M., Esmaeilzadeh S., Karkhah A., Amiri M., Rahmani A., Bakouei F., Nouri H.R. (2020). Elevated expression of IL-18 but not IL-1β gene is associated with NALP3 and AIM2 inflammasome in Polycystic Ovary Syndrome. Gene.

[108] Guo Q.J., Shan J., Xu Y.F., Hu Y.Y., Huo C.L., Song J.Y., Wang C.Q., Zhou H., Yu C.Q., Huang Q. (2020). Pioglitazone Metformin Complex Improves Polycystic Ovary Syndrome Comorbid Psychological Distress via Inhibiting NLRP3 Inflammasome Activation: A Prospective Clinical Study. Mediat. Inflamm..

[109] Hu C., Pang B., Ma Z., Yi H. (2020). Immunophenotypic profiles in polycystic ovary syndrome. Mediat. Inflamm..

[110] Mahajan S., Vita R., Shackelford D., Lane J., Schulten V., Zarebski L., Jespersen M.C., Marcatili P., Nielsen M., Sette A. (2018). Epitope specific antibodies and T cell receptors in the immune epitope database. Front. Immunol..

[111] Rees A.R. (2020). Understanding the human antibody repertoire. MAbs.

[112] Alberto Kölliker Frers R., Otero-Losada M., Inés Herrera M., Porta S., Cosentino V., Kerzberg E., Udovin L., Capani F., Ota Fuchs Q., Athari S.S. (2020). Immune-Mediated Inflammation: Human T CD4 Helper Lymphocyte Diversity and Plasticity in Health and Disease. Cells of the Immune System.

[113] Sbierski-Kind J., Goldeck D., Buchmann N., Spranger J., Volk H.D., Steinhagen-Thiessen E., Pawelec G., Demuth I., Spira D. (2020). T cell phenotypes associated with insulin resistance: Results from the Berlin Aging Study II. Immun. Ageing.

[114] Wu R., Fujii S., Ryan N.K., Van der Hoek K.H., Jasper M.J., Sini I., Robertson S.A., Robker R.L., Norman R.J. (2007). Ovarian leukocyte distribution and cytokine/chemokine mRNA expression in follicular fluid cells in women with polycystic ovary syndrome. Hum. Reprod..

[115] Krishna M.B., Joseph A., Subramaniam A.G., Gupta A., Pillai S.M., Laloraya M. (2015). Reduced tregs in peripheral blood of PCOS patients—A consequence of aberrant Il2 signaling. J. Clin. Endocrinol. Metab..

[116] Ma M., Wang M., Xu F., Hao S. (2022). The Imbalance in Th17 and Treg Cells in Polycystic Ovarian Syndrome Patients with Autoimmune Thyroiditis. Immunol. Investig..

[117] Luan Y.-Y., Zhang L., Peng Y.-Q., Li Y.-Y., Liu R.-X., Yin C.-H. (2022). Immune regulation in polycystic ovary syndrome. Clin. Chim. Acta.

[118] Salvador A.F., de Lima K.A., Kipnis J. (2021). Neuromodulation by the immune system: A focus on cytokines. Nat. Rev. Immunol..

[119] Itoh H., Ueda M., Suzuki M., Kohmura-Kobayashi Y. (2022). Developmental Origins of Metaflammation; A Bridge to the Future Between the DOHaD Theory and Evolutionary Biology. Front. Endocrinol..

[120] Patel S. (2018). Polycystic ovary syndrome (PCOS), an inflammatory, systemic, lifestyle endocrinopathy. J. Steroid Biochem. Mol. Biol..

[121] Pavlov V.A., Wang H., Czura C.J., Friedman S.G., Tracey K.J. (2003). The Cholinergic Anti-inflammatory Pathway: A Missing Link in Neuroimmunomodulation. Mol. Med..

[122] Bellocchi C., Carandina A., Montinaro B., Targetti E., Furlan L., Rodrigues G.D., Tobaldini E., Montano N. (2022). The Interplay between Autonomic Nervous System and Inflammation across Systemic Autoimmune Diseases. Int. J. Mol. Sci..

[123] Ingegnoli F., Buoli M., Antonucci F., Coletto L.A., Esposito C.M., Caporali R. (2020). The Link Between Autonomic Nervous System and Rheumatoid Arthritis: From Bench to Bedside. Front. Med..

[124] del Rey A., Besedovsky H.O., Sorkin E., da Prada M., Arrenbrecht S. (1981). Immunoregulation mediated by the sympathetic nervous system, II. Cell. Immunol..

[125] Martelli D. (2022). The inflammatory reflex reloaded. Brain Behav. Immun..

[126] Zangeneh F.Z., Bagheri M., Naghizadeh M.M. (2015). Hyponeurotrophinemia in Serum of Women with Polycystic Ovary Syndrome as a Low Grade Chronic Inflammation. Open J. Obstet. Gynecol..

[127] Tracey K.J. (2002). The inflammatory reflex. Nature.

[128] Wexler B.C., Dolgin A.E., Tryczynski E.W. (1957). Effects of a bacterial polysaccharide (piromen) on the pituitary-adrenal axis: Adrenal ascorbic acid, cholesterol and histologic alterations. Endocrinology.

[129] Banks W.A., Kastin A.J., Broadwell R. (1995). Passage of cytokines across the blood-brain-barrier. Neuroimmunomodulation.

[130] Buller K.M. (2001). Role of circumventricular organs in pro-inflammatory cytokine-induced activation of the hypothalamic-pituitary-adrenal axis. Clin. Exp. Pharmacol. Physiol..

[131] Berthoud H.R., Neuhuber W.L. (2000). Functional and chemical anatomy of the afferent vagal system. Auton. Neurosci. Basic Clin..

[132] Dag Z.O., Alpua M., Turkel Y., Isik Y. (2015). Autonomic dysfunction in patients with polycystic ovary syndrome. Taiwan. J. Obstet. Gynecol..

[133] Webster J.I., Tonelli L., Sternberg E.M. (2002). Neuroendocrine regulation of immunity. Annu. Rev. Immunol..

[134] Kox M., van Eijk L.T., Zwaag J., van den Wildenberg J., Sweep F.C.G.J., van der Hoeven J.G., Pickkers P. (2014). Voluntary activation of the sympathetic nervous system and attenuation of the innate immune response in humans. Proc. Natl. Acad. Sci. USA.

[135] Sun P., Liu D.G., Ye X.M. (2021). Nicotinic Acetylcholine Receptor α7 Subunit Is an Essential Regulator of Seizure Susceptibility. Front. Neurol..

[136] Shaikh S., Verma H., Yadav N., Jauhari M., Bullangowda J. (2012). Applications of Steroid in Clinical Practice: A Review. ISRN Anesthesiol..

[137] Lansdown A., Rees D.A. (2012). The sympathetic nervous system in polycystic ovary syndrome: A novel therapeutic target?. Clin. Endocrinol..

[138] Chen M.J., Yang W.S., Yang J.H., Chen C.L., Ho H.N., Yang Y.S. (2007). Relationship between androgen levels and blood pressure in young women with polycystic ovary syndrome. Hypertension.

[139] Perciaccante A., Fiorentini A., Valente R., Tubani L. (2007). Polycystic ovary syndrome: Androgens, autonomic nervous system, and hypertension. Hypertension.

[140] Giallauria F., Palomba S., Maresca L., Vuolo L., Tafuri D., Lombardi G., Colao A., Vigorito C., Orio F. (2008). Exercise training improves autonomic function and inflammatory pattern in women with polycystic ovary syndrome (PCOS). Clin. Endocrinol..

[141] Trigunaite A., Dimo J., Jørgensen T.N. (2015). Suppressive effects of androgens on the immune system. Cell. Immunol..

[142] González F. (2012). Inflammation in Polycystic Ovary Syndrome: Underpinning of insulin resistance and ovarian dysfunction. Steroids.

[143] Wagner I.V., Savchuk I., Sahlin L., Kulle A., Klöting N., Dietrich A., Holterhus P.M., Dötsch J., Blüher M., Söder O. (2022). De Novo and Depot-Specific Androgen Production in Human Adipose Tissue: A Source of Hyperandrogenism in Women with Obesity. Obes. Facts.

[144] Velez L.M., Seldin M., Motta A.B. (2021). Inflammation and reproductive function in women with polycystic ovary syndrome. Biol. Reprod..

[145] Xiong Y.L., Liang X.Y., Yang X., Li Y., Wei L.N. (2011). Low-grade chronic inflammation in the peripheral blood and ovaries of women with polycystic ovarian syndrome. Eur. J. Obstet. Gynecol. Reprod. Biol..

[146] Diamanti-Kandarakis E., Alexandraki K., Piperi C., Protogerou A., Katsikis I., Paterakis T., Lekakis J., Panidis D. (2006). Inflammatory and endothelial markers in women with polycystic ovary syndrome. Eur. J. Clin. Investig..

[147] Escobar-Morreale H.F., Luque-Ramírez M., González F. (2011). Circulating inflammatory markers in polycystic ovary syndrome: A systematic review and metaanalysis. Fertil. Steril..

[148] Azhary J.M.K., Harada M., Takahashi N., Nose E., Kunitomi C., Koike H., Hirata T., Hirota Y., Koga K., Wada-Hiraike O. (2019). Endoplasmic reticulum stress activated by androgen enhances apoptosis of granulosa cells via induction of death receptor 5 in PCOS. Endocrinology.

[149] Koike H., Harada M., Kusamoto A., Xu Z., Tanaka T., Sakaguchi N., Kunitomi C., Azhary J.M.K., Takahashi N., Urata Y. (2023). Roles of endoplasmic reticulum stress in the pathophysiology of polycystic ovary syndrome. Front. Endocrinol..

[150] Li Y., Zheng Q., Sun D., Cui X., Chen S., Bulbul A., Liu S., Yan Q. (2019). Dehydroepiandrosterone stimulates inflammation and impairs ovarian functions of polycystic ovary syndrome. J. Cell. Physiol..

[151] Zhai Y., Pang Y. (2022). Systemic and ovarian inflammation in women with polycystic ovary syndrome. J. Reprod. Immunol..

[152] Rahman M.S., Hossain K.S., Das S., Kundu S., Adegoke E.O., Rahman M.A., Hannan M.A., Uddin M.J., Pang M.G. (2021). Role of insulin in health and disease: An update. Int. J. Mol. Sci..

[153] Watanabe M., Hayasaki H., Tamayama T., Shimada M. (1998). Histologic distribution of insulin and glucagon receptors. Braz. J. Med. Biol. Res..

[154] Khalid M., Alkaabi J., Khan M.A.B., Adem A. (2021). Insulin signal transduction perturbations in insulin resistance. Int. J. Mol. Sci..

[155] Petersen M.C., Shulman G.I. (2018). Mechanisms of insulin action and insulin resistance. Physiol. Rev..

[156] Zhou M.S., Wang A., Yu H. (2014). Link between insulin resistance and hypertension: What is the evidence from evolutionary biology?. Diabetol. Metab. Syndr..

[157] Sun Q., Li J., Gao F. (2014). New insights into insulin: The anti-inflammatory effect and its clinical relevance. World J. Diabetes.

[158] Chang Y.W., Hung L.C., Chen Y.C., Wang W.H., Lin C.Y., Tzeng H.H., Suen J.L., Chen Y.H. (2021). Insulin Reduces Inflammation by Regulating the Activation of the NLRP3 Inflammasome. Front. Immunol..

[159] Fernández-Real J.M., Ricart W. (1999). Insulin resistance and inflammation in an evolutionary perspective: The contribution of cytokine genotype/phenotype to thriftiness. Diabetologia.

[160] Wan Y.Y. (2010). Regulatory T cells: Immune suppression and beyond. Cell. Mol. Immunol..

[161] Jacobse J., Li J., Rings E.H.H.M., Samsom J.N., Goettel J.A. (2021). Intestinal Regulatory T Cells as Specialized Tissue-Restricted Immune Cells in Intestinal Immune Homeostasis and Disease. Front. Immunol..

[162] Makhijani P., Basso P.J., Chan Y.T., Chen N., Baechle J., Khan S., Furman D., Tsai S., Winer D. (2023). Regulation of the immune system by the insulin receptor in health and disease. Front. Endocrinol..

[163] Cassar S., Misso M.L., Hopkins W.G., Shaw C.S., Teede H.J., Stepto N.K. (2016). Insulin resistance in polycystic ovary syndrome: A systematic review and meta-analysis of euglycaemic-hyperinsulinaemic clamp studies. Hum. Reprod..

[164] Stepto N.K., Cassar S., Joham A.E., Hutchison S.K., Harrison C.L., Goldstein R.F., Teede H.J. (2013). Women with polycystic ovary syndrome have intrinsic insulin resistance on euglycaemic-hyperinsulaemic clamp. Hum. Reprod..

[165] Katic M., Kahn C.R. (2005). The role of insulin and IGF-1 signaling in longevity. Cell. Mol. Life Sci..

[166] Cibula D. (2004). Is insulin resistance an essential component of PCOS? The influence of confounding factors. Hum. Reprod..

[167] Plomin R., Haworth C.M.A., Davis O.S.P. (2009). Common disorders are quantitative traits. Nat. Rev. Genet..

[168] Tam C.S., Xie W., Johnson W.D., Cefalu W.T., Redman L.M., Ravussin E. (2012). Defining insulin resistance from hyperinsulinemic-euglycemic clamps. Diabetes Care.

[169] Dunaif A., Segal K.R., Futterweit W., Dobrjansky A. (1989). Profound peripheral insulin resistance, independent of obesity, in polycystic ovary syndrome. Diabetes.

[170] Teede H.J., Hutchison S.K., Zoungas S. (2007). The management of insulin resistance in polycystic ovary syndrome. Trends Endocrinol. Metab..

[171] Toosy S., Sodi R., Pappachan J.M. (2018). Lean polycystic ovary syndrome (PCOS): An evidence-based practical approach. J. Diabetes Metab. Disord..

[172] Morciano A., Romani F., Sagnella F., Scarinci E., Palla C., Moro F., Tropea A., Policola C., Della Casa S., Guido M. (2014). Assessment of insulin resistance in lean women with polycystic ovary syndrome. Fertil. Steril..

[173] Gorjão R., Takahashi H.K., Pan J.A., Massao Hirabara S. (2012). Molecular mechanisms involved in inflammation and insulin resistance in chronic diseases and possible interventions. J. Biomed. Biotechnol..

[174] Fernandez M., Murillo A. (2022). Dietary Treatments to Reduce Insulin Resistance and Inflammation in Type-2 Diabetic Patients. Med. Res. Arch..

[175] Tsatsoulis A., Mantzaris M.D., Sofia B., Andrikoula M. (2012). Insulin resistance: An adaptive mechanism becomes maladaptive in the current environment—An evolutionary perspective. Metabolism.

[176] Wensveen F.M., Šestan M., Turk Wensveen T., Polić B. (2019). ‘Beauty and the beast’ in infection: How immune–endocrine interactions regulate systemic metabolism in the context of infection. Eur. J. Immunol..

[177] Wang P., Mariman E.C.M. (2008). Insulin resistance in an energy-centered perspective. Physiol. Behav..

[178] Kampmann U., Knorr S., Fuglsang J., Ovesen P. (2019). Determinants of Maternal Insulin Resistance during Pregnancy: An Updated Overview. J. Diabetes Res..

[179] Sprague J.E., Gandica R., Kelsey M.M. (2020). Insulin Resistance in Puberty.

[180] Straub R.H., Cutolo M., Buttgereit F., Pongratz G. (2010). Energy regulation and neuroendocrine-immune control in chronic inflammatory diseases. J. Intern. Med..

[181] Rabasa C., Dickson S.L. (2016). Impact of stress on metabolism and energy balance. Curr. Opin. Behav. Sci..

[182] Ieronymaki E., Daskalaki M.G., Lyroni K., Tsatsanis C. (2019). Insulin signaling and insulin resistance facilitate trained immunity in macrophages through metabolic and epigenetic changes. Front. Immunol..

[183] Chaudhari A.P., Mazumdar K., Deepak P. (2018). Anxiety, Depression, and Quality of Life in Women with Polycystic Ovarian Syndrome. Indian J. Psychol. Med..

[184] Soeters M.R., Soeters P.B. (2012). The evolutionary benefit of insulin resistance. Clin. Nutr..

[185] Soeters M.R., Sauerwein H.P., Dubbelhuis P.F., Groener J.E., Ackermans M.T., Fliers E., Aerts J.M., Serlie M.J. (2008). Muscle adaptation to short-term fasting in healthy lean humans. J. Clin. Endocrinol. Metab..

[186] Horita S., Seki G., Yamada H., Suzuki M., Koike K., Fujita T. (2011). Insulin resistance, obesity, hypertension, and renal sodium transport. Int. J. Hypertens..

[187] Velloso L.A., Folli F., Perego L., Saad M.J.A. (2006). The multi-faceted cross-talk between the insulin and angiotensin II signaling systems. Diabetes. Metab. Res. Rev..

[188] Zhou M.S., Schulman I.H., Raij L. (2010). Vascular inflammation, insulin resistance, and endothelial dysfunction in salt-sensitive hypertension: Role of nuclear factor kappa B activation. J. Hypertens..

[189] Ehrmann D.A., Liljenquist D.R., Kasza K., Azziz R., Legro R.S., Ghazzi M.N., Aronoff S., Bernstein R., Bodenner D., Braithwaite S. (2006). Prevalence and predictors of the metabolic syndrome in women with polycystic ovary syndrome. J. Clin. Endocrinol. Metab..

[190] Sonagra A.D. (2014). Normal Pregnancy- A State of Insulin Resistance. J. Clin. Diagnostic Res..

[191] Catalano P.M., Huston L., Amini S.B., Kalhan S.C. (1999). Longitudinal changes in glucose metabolism during pregnancy in obese women with normal glucose tolerance and gestational diabetes mellitus. Am. J. Obstet. Gynecol..

[192] Jayabalan N., Nair S., Nuzhat Z., Rice G.E., Zuñiga F.A., Sobrevia L., Leiva A., Sanhueza C., Gutiérrez J.A., Lappas M. (2017). Cross talk between adipose tissue and placenta in obese and gestational diabetes mellitus pregnancies via exosomes. Front. Endocrinol..

[193] Koren O., Goodrich J.K., Cullender T.C., Spor A., Laitinen K., Kling Bäckhed H., Gonzalez A., Werner J.J., Angenent L.T., Knight R. (2012). Host remodeling of the gut microbiome and metabolic changes during pregnancy. Cell.

[194] Li X., Liu X., Zuo Y., Gao J., Liu Y., Zheng W. (2021). The risk factors of gestational diabetes mellitus in patients with polycystic ovary syndrome: What should we care. Medicine.

[195] Yan Q., Qiu D., Liu X., Xing Q., Liu R., Hu Y. (2022). The incidence of gestational diabetes mellitus among women with polycystic ovary syndrome: A meta-analysis of longitudinal studies. BMC Pregnancy Childbirth.

[196] Noctor E. (2015). Type 2 diabetes after gestational diabetes: The influence of changing diagnostic criteria. World J. Diabetes.

[197] Vounzoulaki E., Khunti K., Abner S.C., Tan B.K., Davies M.J., Gillies C.L. (2020). Progression to type 2 diabetes in women with a known history of gestational diabetes: Systematic review and meta-analysis. BMJ.

[198] Bonora E., Trombetta M., Dauriz M., Travia D., Cacciatori V., Brangani C., Negri C., Perrone F., Pichiri I., Stoico V. (2020). Chronic complications in patients with newly diagnosed type 2 diabetes: Prevalence and related metabolic and clinical features: The Verona Newly Diagnosed Type 2 Diabetes Study (VNDS) 9. BMJ Open Diabetes Res. Care.

[199] Bolton A. (2021). International Diabetes Federation Diabetes Atlas.

[200] Yang J., Qian F., Chavarro J.E., Ley S.H., Tobias D.K., Yeung E., Hinkle S.N., Bao W., Li M., Liu A. (2022). Modifiable risk factors and long term risk of type 2 diabetes among individuals with a history of gestational diabetes mellitus: Prospective cohort study. BMJ.

[201] Patel S. (2017). Disruption of aromatase homeostasis as the cause of a multiplicity of ailments: A comprehensive review. J. Steroid Biochem. Mol. Biol..

[202] Evans S.F., Hull M.L., Hutchinson M.R., Rolan P.E. (2021). Androgens, Endometriosis and Pain. Front. Reprod. Health.

[203] Diamond M.P., Grainger D., Diamond M.C., Sherwin R.S., Defronzo R.A. (1998). Effects of methyltestosterone on insulin secretion and sensitivity in women. J. Clin. Endocrinol. Metab..

[204] Polderman K.H., Gooren L.J., Asscheman H. (1994). Induction of Insulin Resistance by Androgens and Estrogens. J. Clin. Endocrinol. Metab..

[205] Skinner M.K., Schmidt M., Savenkova M.I., Sadler-Riggleman I., Nilsson E.E. (2008). Regulation of granulosa and theca cell transcriptomes during ovarian antral follicle development. Mol. Reprod. Dev..

[206] Clegg D.J. (2012). Minireview: The year in review of estrogen regulation of metabolism. Mol. Endocrinol..

[207] Stocco C. (2008). Aromatase expression in the ovary: Hormonal and molecular regulation. Steroids.

[208] Armanini D., Boscaro M., Bordin L., Sabbadin C. (2022). Controversies in the Pathogenesis, Diagnosis and Treatment of PCOS: Focus on Insulin Resistance, Inflammation, and Hyperandrogenism. Int. J. Mol. Sci..

[209] Di Nardo G., Zhang C., Marcelli A.G., Gilardi G. (2021). Molecular and structural evolution of cytochrome p450 aromatase. Int. J. Mol. Sci..

[210] Simeon S., Spjuth O., Lapins M., Nabu S., Anuwongcharoen N., Prachayasittikul V., Wikberg J.E.S., Nantasenamat C. (2016). Origin of aromatase inhibitory activity via proteochemometric modeling. PeerJ.

[211] Conley A., Mapes S., Jo Corbin C., Greger D., Graham S. (2002). Structural determinants of aromatase cytochrome p450 inhibition in substrate recognition site-1. Mol. Endocrinol..

[212] Jin Y., Choi Y.J., Heo K., Park S.J. (2021). Melatonin as an oncostatic molecule based on its anti-aromatase role in breast cancer. Int. J. Mol. Sci..

[213] Cheng J.C., Fang L., Li Y., Wang S., Li Y., Yan Y., Jia Q., Wu Z., Wang Z., Han X. (2020). Melatonin stimulates aromatase expression and estradiol production in human granulosa-lutein cells: Relevance for high serum estradiol levels in patients with ovarian hyperstimulation syndrome. Exp. Mol. Med..

[214] Kim J.Y., Han E.H., Kim H.G., Oh K.N., Kim S.K., Lee K.Y., Jeong H.G. (2010). Bisphenol A-induced aromatase activation is mediated by cyclooxygenase-2 up-regulation in rat testicular Leydig cells. Toxicol. Lett..

[215] Maia H., Haddad C., Pinheiro N., Casoy J. (2013). The effect of oral contraceptives on aromatase and Cox-2 expression in the endometrium of patients with idiopathic menorrhagia or adenomyosis. Int. J. Womens. Health.

[216] Wang C., Mäkelä T., Hase T., Adlercreutz H., Kurzer M.S. (1994). Lignans and flavonoids inhibit aromatase enzyme in human preadipocytes. J. Steroid Biochem. Mol. Biol..

[217] Singh S., Awasthi M., Pandey V.P., Dwivedi U.N. (2017). Plant derived anti-cancerous secondary metabolites as multipronged inhibitor of COX, Topo, and aromatase: Molecular modeling and dynamics simulation analyses. J. Biomol. Struct. Dyn..

[218] Richard S., Moslemi S., Sipahutar H., Benachour N., Seralini G.E. (2005). Differential effects of glyphosate and roundup on human placental cells and aromatase. Environ. Health Perspect..

[219] Zarn J.A., Brüschweiler B.J., Schlatter J.R. (2003). Azole fungicides affect mammalian steroidogenesis by inhibiting sterol 14α-demethylase and aromatase. Environ. Health Perspect..

[220] Rojas J., Chávez-Castillo M., Torres W., Arraiz N., Cabrera M. (2014). Metformin in Cancer: Chemical Pathways for Tumoral Control Independent of Amp-Dependent Kinase. J. Endocrinol. Diabetes Obes..

[221] Biegon A., Alia-Klein N., Fowler J.S. (2012). Potential contribution of aromatase inhibition to the effects of nicotine and related compounds on the brain. Front. Pharmacol..

[222] Lee S.H., Park S.Y., Choi C.S. (2022). Insulin Resistance: From Mechanisms to Therapeutic Strategies. Diabetes Metab. J..

[223] Baillargeon J.P., Nestler J.E. (2006). Commentary: Polycystic ovary syndrome: A syndrome of ovarian hypersensitivity to insulin?. J. Clin. Endocrinol. Metab..

[224] Zhao H., Zhang J., Cheng X., Nie X., He B. (2023). Insulin resistance in polycystic ovary syndrome across various tissues: An updated review of pathogenesis, evaluation, and treatment. J. Ovarian Res..

[225] Bremer A.A., Miller W.L. (2008). The serine phosphorylation hypothesis of polycystic ovary syndrome: A unifying mechanism for hyperandrogenemia and insulin resistance. Fertil. Steril..

[226] Ibáñez L., Potau N., Zampolli M., Riqué S., Saenger P., Carrascosa A. (1997). Hyperinsulinemia and decreased insulin-like growth factor-binding protein-1 are common features in prepubertal and pubertal girls with a history of premature pubarche. J. Clin. Endocrinol. Metab..

[227] Soldani R., Cagnacci A., Yen S.S.C. (1994). Insulin insulin-like growth factor I (IGF-I) and IGF-II enhance basal and gonadotrophin-releasing hormone-stimulated luteinizing hormone release from rat anterior pituitary cells in vitro. Eur. J. Endocrinol..

[228] Nestler J.E., Powers L.P., Matt D.W., Steingold K.A., Plymate S.R., Rittmaster R.S., Clore J.N., Blackard W.G. (1991). A direct effect of hyperinsulinemia on serum sex hormone-binding globulin levels in obese women with the polycystic ovary syndrome. J. Clin. Endocrinol. Metab..

[229] Chan O., Inouye K., Akirav E., Park E., Riddell M.C., Vranic M., Matthews S.G. (2005). Insulin alone increases hypothalamo-pituitary-adrenal activity, and diabetes lowers peak stress responses. Endocrinology.

[230] Kanbour S.A., Dobs A.S. (2022). Hyperandrogenism in Women with Polycystic Ovarian Syndrome: Pathophysiology and Controversies. Androgens.

[231] Pateguana N.B., Janes A. (2019). The contribution of hyperinsulinemia to the hyperandrogenism of polycystic ovary syndrome. J. Insul. Resist..

[232] Vyrides A.A., El Mahdi E., Giannakou K. (2022). Ovulation induction techniques in women with polycystic ovary syndrome. Front. Med..

[233] Panth N., Gavarkovs A., Tamez M., Mattei J. (2018). The Influence of Diet on Fertility and the Implications for Public Health Nutrition in the United States. Front. Public Health.

[234] Elnashar A.M. (2011). The role of metformin in ovulation induction: Current status. Middle East Fertil. Soc. J..

[235] Nehir Aytan A., Bastu E., Demiral I., Bulut H., Dogan M., Buyru F. (2016). Relationship between hyperandrogenism, obesity, inflammation and polycystic ovary syndrome. Gynecol. Endocrinol..

[236] West-Eberhard M.J. (2019). Nutrition, the visceral immune system, and the evolutionary origins of pathogenic obesity. Proc. Natl. Acad. Sci. USA.

[237] Ottaviani E., Malagoli D., Franceschi C. (2011). The evolution of the adipose tissue: A neglected enigma. Gen. Comp. Endocrinol..

[238] Kuzawa C.W. (1998). Adipose Tissue in Human Infancy and Childhood: An Evolutionary Perspective. Yearb. Phys. Anthropol..

[239] Parra-Peralbo E., Talamillo A., Barrio R. (2021). Origin and Development of the Adipose Tissue, a Key Organ in Physiology and Disease. Front. Cell Dev. Biol..

[240] Ibrahim M.M. (2010). Subcutaneous and visceral adipose tissue: Structural and functional differences. Obes. Rev..

[241] Spritzer P.M., Lecke S.B., Satler F., Morsch D.M. (2015). Adipose tissue dysfunction, adipokines, and low-grade chronic inflammation in polycystic ovary syndrome. Reproduction.

[242] Scheja L., Heeren J. (2019). The endocrine function of adipose tissues in health and cardiometabolic disease. Nat. Rev. Endocrinol..

[243] Choe S.S., Huh J.Y., Hwang I.J., Kim J.I., Kim J.B. (2016). Adipose tissue remodeling: Its role in energy metabolism and metabolic disorders. Front. Endocrinol..

[244] Pawłowski B., Żelaźniewicz A. (2021). The evolution of perennially enlarged breasts in women: A critical review and a novel hypothesis. Biol. Rev..

[245] Tan C.Y., Vidal-Puig A. (2008). Adipose tissue expandability: The metabolic problems of obesity may arise from the inability to become more obese. Biochem. Soc. Trans..

[246] Villa J., Pratley R.E. (2011). Adipose tissue dysfunction in polycystic ovary syndrome. Curr. Diab. Rep..

[247] Echiburú B., Pérez-Bravo F., Galgani J.E., Sandoval D., Saldías C., Crisosto N., Maliqueo M., Sir-Petermann T. (2018). Enlarged adipocytes in subcutaneous adipose tissue associated to hyperandrogenism and visceral adipose tissue volume in women with polycystic ovary syndrome. Steroids.

[248] Arpaci D., Gurkan Tocoglu A., Yilmaz S., Ergenc H., Tamer A., Keser N., Gunduz H. (2015). The relationship between epicardial fat tissue thickness and visceral adipose tissue in lean patients with polycystic ovary syndrome. J. Ovarian Res..

[249] Glueck C.J., Goldenberg N. (2019). Characteristics of obesity in polycystic ovary syndrome: Etiology, treatment, and genetics. Metabolism.

[250] Kałużna M., Czlapka-Matyasik M., Bykowska-Derda A., Moczko J., Ruchala M., Ziemnicka K. (2021). Indirect predictors of visceral adipose tissue in women with polycystic ovary syndrome: A comparison of methods. Nutrients.

[251] Jones G.W., Hill D.G., Jones S.A. (2016). Understanding immune cells in tertiary lymphoid organ development: It is all starting to come together. Front. Immunol..

[252] Dixon L.J., Barnes M., Tang H., Pritchard M.T., Nagy L.E. (2013). Kupffer cells in the liver. Compr. Physiol..

[253] Parker J., O’Brien C., Gersh F.L. (2021). Developmental origins and transgenerational inheritance of polycystic ovary syndrome. Aust. N. Z. J. Obstet. Gynaecol..

[254] Davenport E.R., Sanders J.G., Song S.J., Amato K.R., Clark A.G., Knight R. (2017). The human microbiome in evolution. BMC Biol..

[255] Rizk M.G., Thackray V.G. (2021). Intersection of Polycystic Ovary Syndrome and the Gut Microbiome. J. Endocr. Soc..

[256] Valdes A.M., Walter J., Segal E., Spector T.D. (2018). Role of the gut microbiota in nutrition and health. BMJ.

[257] Martinez J.E., Kahana D.D., Ghuman S., Wilson H.P., Wilson J., Kim S.C.J., Lagishetty V., Jacobs J.P., Sinha-Hikim A.P., Friedman T.C. (2021). Unhealthy Lifestyle and Gut Dysbiosis: A Better Understanding of the Effects of Poor Diet and Nicotine on the Intestinal Microbiome. Front. Endocrinol..

[258] Degruttola A.K., Low D., Mizoguchi A., Mizoguchi E. (2016). Current understanding of dysbiosis in disease in human and animal models. Inflamm. Bowel Dis..

[259] Shahi S.K., Ghimire S., Lehman P., Mangalam A.K. (2022). Obesity induced gut dysbiosis contributes to disease severity in an animal model of multiple sclerosis. Front. Immunol..

[260] Moya A., Ferrer M. (2016). Functional Redundancy-Induced Stability of Gut Microbiota Subjected to Disturbance. Trends Microbiol..

[261] Zhao X., Jiang Y., Xi H., Chen L., Feng X. (2020). Exploration of the Relationship between Gut Microbiota and Polycystic Ovary Syndrome (PCOS): A Review. Geburtshilfe Frauenheilkd..

[262] Yilmaz B., Terekeci H., Sandal S., Kelestimur F. (2020). Endocrine disrupting chemicals: Exposure, effects on human health, mechanism of action, models for testing and strategies for prevention. Rev. Endocr. Metab. Disord..

[263] Fabozzi G., Rebuzzini P., Cimadomo D., Allori M., Franzago M., Stuppia L., Garagna S., Ubaldi F.M., Zuccotti M., Rienzi L. (2022). Endocrine-Disrupting Chemicals, Gut Microbiota, and Human (In) Fertility—It Is Time to Consider the Triad. Cells.

[264] Bellingham M., Sharpe R. (2013). Chemical Exposures During Pregnancy: Dealing with Potential, but Unproven, Risks to Child Health. R. Coll. Obstet. Gynaecol..

[265] Di Renzo G.C., Conry J.A., Blake J., Defrancesco M.S., Denicola N., Martin J.N., McCue K.A., Richmond D., Shah A., Sutton P. (2015). International Federation of Gynecology and Obstetrics opinion on reproductive health impacts of exposure to toxic environmental chemicals. Int. J. Gynecol. Obstet..

[266] Gore A.C., Chappell V.A., Fenton S.E., Flaws J.A., Nadal A., Prins G.S., Toppari J., Zoeller R.T. (2015). EDC-2: The Endocrine Society’s Second Scientific Statement on Endocrine-Disrupting Chemicals. Endocr. Rev..

[267] Liu Z., Lu Y., Zhong K., Wang C., Xu X. (2022). The associations between endocrine disrupting chemicals and markers of inflammation and immune responses: A systematic review and meta-analysis. Ecotoxicol. Environ. Saf..

[268] Alonso-Magdalena P., Morimoto S., Ripoll C., Fuentes E., Nadal A. (2006). The estrogenic effect of bisphenol a disrupts pancreatic β-cell function in vivo and induces insulin resistance. Environ. Health Perspect..

[269] Wang Y., Zhu Q., Dang X., He Y., Li X., Sun Y. (2017). Local effect of bisphenol A on the estradiol synthesis of ovarian granulosa cells from PCOS. Gynecol. Endocrinol..

[270] Sun Y., Gao S., Ye C., Zhao W. (2023). Gut microbiota dysbiosis in polycystic ovary syndrome: Mechanisms of progression and clinical applications. Front. Cell. Infect. Microbiol..

[271] Ananthasubramanian P., Ananth S., Kumaraguru S., Barathi S., Santosh W., Vasantharekha R. (2021). Associated Effects of Endocrine Disrupting Chemicals (EDCs) on Neuroendocrine Axes and Neurotransmitter Profile in Polycystic Ovarian Syndrome Condition. Proc. Zool. Soc..

[272] Gupta R., Kumar P., Fahmi N., Garg B., Dutta S., Sachar S., Matharu A.S., Vimaleswaran K.S. (2020). Endocrine disruption and obesity: A current review on environmental obesogens. Curr. Res. Green Sustain. Chem..

[273] Aydemir D., Ulusu N.N. (2023). The possible role of the endocrine disrupting chemicals on the premature and early menopause associated with the altered oxidative stress metabolism. Front. Endocrinol..

[274] Ravichandran G., Lakshmanan D.K., Raju K., Elangovan A., Nambirajan G., Devanesan A.A., Thilagar S. (2019). Food advanced glycation end products as potential endocrine disruptors: An emerging threat to contemporary and future generation. Environ. Int..

[275] Bansal A., Henao-Mejia J., Simmons R.A. (2018). Immune system: An emerging player in mediating effects of endocrine disruptors on metabolic health. Endocrinology.

[276] Tudurí E., Marroqui L., Dos Santos R.S., Quesada I., Fuentes E., Alonso-Magdalena P. (2018). Timing of exposure and Bisphenol-A: Implications for diabetes development. Front. Endocrinol..

[277] Resnik D.B. (2004). The precautionary principle and medical decision making. J. Med. Philos..

[278] Schug T.T., Johnson A.F., Birnbaum L.S., Colborn T., Guillette L.J., Crews D.P., Collins T., Soto A.M., Vom Saal F.S., McLachlan J.A. (2016). Minireview: Endocrine disruptors: Past lessons and future directions. Mol. Endocrinol..

[279] More S., Bampidis V., Benford D., Bragard C., Halldorsson T., Hernández-Jerez A., Hougaard Bennekou S., Koutsoumanis K., Lambré C., Machera K. (2021). Guidance on risk assessment of nanomaterials to be applied in the food and feed chain: Human and animal health. EFSA J..

[280] Rutkowska A.Z., Diamanti-Kandarakis E. (2016). Polycystic ovary syndrome and environmental toxins. Fertil. Steril..

[281] Jala A., Varghese B., Kaur G., Rajendiran K., Dutta R., Adela R., Borkar R.M. (2022). Implications of endocrine-disrupting chemicals on polycystic ovarian syndrome: A comprehensive review. Environ. Sci. Pollut. Res..

[282] Jozkowiak M., Piotrowska-Kempisty H., Kobylarek D., Gorska N., Mozdziak P., Kempisty B., Rachon D., Spaczynski R.Z. (2023). Endocrine Disrupting Chemicals in Polycystic Ovary Syndrome: The Relevant Role of the Theca and Granulosa Cells in the Pathogenesis of the Ovarian Dysfunction. Cells.

[283] Parker J. (2015). A new hypothesis for the mechanism of glyphosate induced intestinal permeability in the pathogenesis of polycystic ovary syndrome. J. Australas. Coll. Nutr. Environ. Med..

[284] Kumar M., Sarma D.K., Shubham S., Kumawat M., Verma V., Prakash A., Tiwari R. (2020). Environmental Endocrine-Disrupting Chemical Exposure: Role in Non-Communicable Diseases. Front. Public Health.

[285] Mitro S.D., Johnson T., Zota A.R. (2015). Cumulative Chemical Exposures During Pregnancy and Early Development. Curr. Environ. Health Rep..

[286] Starling A.P., Adgate J.L., Hamman R.F., Kechris K., Calafat A.M., Ye X., Dabelea D. (2017). Perfluoroalkyl substances during pregnancy and offspring weight and adiposity at birth: Examining mediation by maternal fasting glucose in the healthy start study. Environ. Health Perspect..

[287] Rosenfeld C.S. (2021). Transcriptomics and Other Omics Approaches to Investigate Effects of Xenobiotics on the Placenta. Front. Cell Dev. Biol..

[288] Hewlett M., Chow E., Aschengrau A., Mahalingaiah S. (2017). Prenatal Exposure to Endocrine Disruptors: A Developmental Etiology for Polycystic Ovary Syndrome. Reprod. Sci..

[289] Corbett G.A., Lee S., Woodruff T.J., Hanson M., Hod M., Charlesworth A.M., Giudice L., Conry J., McAuliffe F.M. (2022). Nutritional interventions to ameliorate the effect of endocrine disruptors on human reproductive health: A semi-structured review from FIGO. Int. J. Gynecol. Obstet..

[290] Piazza M.J., Urbanetz A.A. (2019). Environmental toxins and the impact of other endocrine disrupting chemicals in women’s reproductive health. J. Bras. Reprod. Assist..

[291] Hanahan D., Weinberg R.A. (2000). The Hallmarks of Cancer. Cell.

[292] López-Otín C., Blasco M.A., Partridge L., Serrano M., Kroemer G. (2013). The hallmarks of aging. Cell.

